# FluxAtlas is a pan-cancer metabolic flux atlas that identifies tumor-selective dependencies and prognostic biomarkers

**DOI:** 10.1016/j.celrep.2026.118029

**Published:** 2026-09-24

**Authors:** Manas Kohli, Aurélien Tripp, Anastasiia Shcherbakova, George Poulogiannis

**Affiliations:** 1 Signalling and Cancer Metabolism Laboratory, Division of Cell and Molecular Biology, The Institute of Cancer Research, 237 Fulham Road, London SW3 6JB, UK; 2 Research Software Engineering Group, The Institute of Cancer Research, 237 Fulham Road, London SW3 6JB, UK

**Keywords:** metabolic reprogramming, cancer, metabolic flux, FluxAtlas, genome-scale metabolic modeling, therapeutic targets, prognostic biomarkers

## Abstract

Metabolic reprogramming is a hallmark of cancer, yet dynamic metabolic flux has been difficult to study systematically. Here, we present FluxAtlas, a pan-cancer atlas of metabolic flux generated from genome-scale metabolic modeling of over 10,000 tumors across 28 The Cancer Genome Atlas (TCGA) cancer types. By integrating enzyme constraints and nutrient diffusion limits, we reveal conserved and tissue-specific metabolic rewiring, including alterations in bile acid recycling, urea metabolism, and amino acid biosynthesis. We identify a bile acid-associated program that remodels glutathione homeostasis and drives gastrointestinal-specific lipid metabolism. Comparisons with matched normal models uncover tumor-selective metabolic dependencies, such as increased reliance of renal cancers on *de novo* purine synthesis. Under nutrient limitation, modeling predicts convergence on glutamine-dependent aspartate synthesis while preserving tissue-specific metabolic states. Machine learning models based on fluxomics predict patient survival and highlight biotin uptake as a prognostic biomarker. FluxAtlas defines the functional metabolic landscape of human cancer and is accessible at https://software.icr.ac.uk/app/flux-atlas.

## Highlights

• FluxAtlas maps metabolic flux in over 10,000 tumors across 28 cancer types
• Enzyme and nutrient constraints reveal how the microenvironment shapes tumor flux
• Bile acid metabolism and renal cancer purine synthesis emerge as key liabilities
• Flux-based models predict survival and highlight biotin uptake as a biomarker

## Graphical abstract

**Figure:**
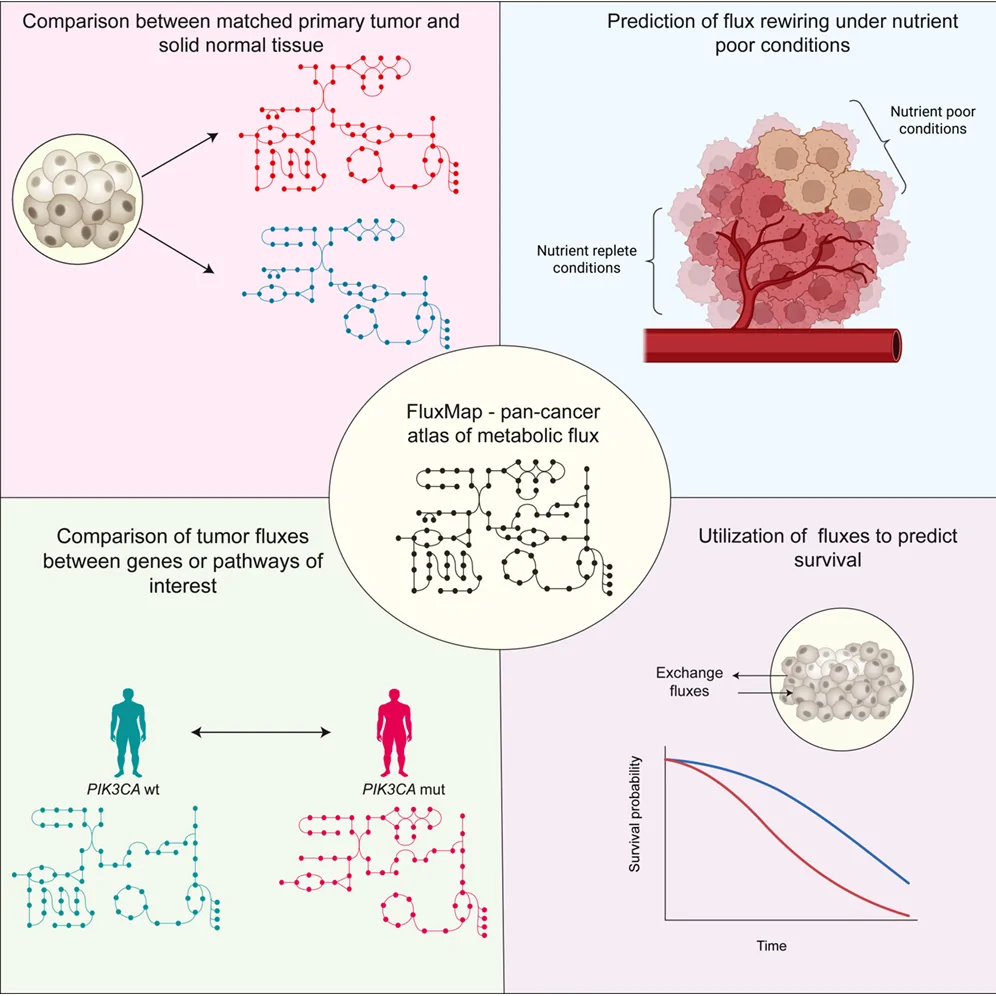

## Teaser

Kohli et al. apply enzyme- and nutrient-constrained genome-scale modeling to more than 10,000 tumors across 28 cancer types, defining a pan-cancer metabolic-flux landscape. This analysis reveals tissue-specific metabolic adaptations and vulnerabilities, including bile acid-linked redox and lipid rewiring, renal-specific purine dependence, and flux-based survival predictors, such as biotin uptake.

## Introduction

Metabolic reprogramming is a critical hallmark of cancer.^1^ To support proliferation, survival, and migration, tumor cells undergo metabolic adaptations, often within nutrient-depleted environments.^2^ This metabolic shift in tumors presents significant therapeutic and diagnostic opportunities.^3^ Indeed, clinical studies have shown a link between tumor metabolic phenotypes and patient outcomes. For instance, triple-negative breast cancers that are highly glycolytic correlate with a poorer response to neoadjuvant chemotherapy and a greater risk of recurrence.^4^

Metabolomics approaches, including label free and stable isotope-assisted methods, have been used to study cancer metabolism,^5^ but their integration into precision medicine has lagged behind other omics technologies.^6^ Many cancer studies prioritize genetic and transcriptomic alterations because, unlike other omics technologies, metabolomics lacks a single experimental platform capable of comprehensively profiling the metabolome.^7^ Consequently, transcriptomic approaches are often used as surrogates to study metabolic reprogramming,^8^^,^^9^^,^^10^ typically using statistical approaches such as ssGSEA,^11^ AUCell,^12^ and *Z* scores of normalized mRNA expression to estimate metabolic activity. However, these approaches have limited power for small metabolic subsystems^13^ and tend to analyze pathways in isolation, missing broader metabolic network adaptations. In addition, many metabolic targets identified in this manner often have poor specificity, given that surrounding normal tissues also, in part, rely on the same metabolic pathways.^14^

Given the vast chemical space of the cellular metabolome, techniques that can examine the entire cellular metabolome are needed. To interrogate entire metabolic networks at a genome-wide scale, genome-scale metabolic models (GEMs), which encapsulate all of an organism’s stoichiometrically balanced metabolic reactions, have been utilized.^15^ GEMs provide a framework for interrogating entire metabolic networks by integrating omics data to generate cell- or patient-specific models. Flux balance analysis (FBA) can then predict genome-wide metabolic flux^16^ and gene essentiality, providing insight into metabolic dependencies and adaptation to perturbations.^17^

Here, we developed a pan-cancer atlas of personalized metabolic flux states to systematically investigate how tumor metabolic programs are shaped by tissue context, genetic alterations, and nutrient environments. Although individual metabolic dependencies have been identified across cancer types, distinguishing universal from context-specific metabolic phenotypes requires systematic comparison across diverse tumor lineages. By integrating transcriptomic, enzymatic, and physiologically informed nutrient constraints into GEMs across more than 10,000 samples from The Cancer Genome Atlas (TCGA)^18^ spanning 28 tumor types, we aimed to move beyond characterizing individual metabolic pathways and instead identify functional metabolic behaviors that emerge across distinct biological contexts. Using this framework, we identify shared and tissue-restricted metabolic adaptations acquired during tumor development, uncover regulatory mechanisms associated with altered metabolic states, and reveal context-specific vulnerabilities driven by both tumor lineage and oncogenic alterations. We further demonstrate how this atlas can be used to investigate metabolic adaptation to nutrient availability and evaluate whether flux-derived metabolic states provide prognostic information associated with patient survival. Together, our study provides a functional resource for contextualizing tumor metabolism across cancers and prioritizing metabolic phenotypes for experimental investigation.

## Results

### Modeling cancer metabolism: A pan-cancer FBA approach

We generated a pan-cancer resource of personalized metabolic models to compare functional metabolic states across tumor lineages, genetic backgrounds, and environmental conditions. Using the rigorously curated Human1 metabolic reconstruction (13,082 reactions and 8,378 metabolites),^19^ we generated patient-specific GEMs from TCGA transcriptomes spanning 28 tumor types using tINIT.^20^ Additionally, where available, we created GEMs for matched normal tissue present in TCGA and compared these to their corresponding primary tumor GEMs (Figure S1). Although GEMs and flux distributions were generated for all 28 tumor types (Figure S2C), analyses requiring direct comparison between tumor and normal metabolic states were restricted to the 16 tumor types with at least 10 corresponding normal tissue samples (Figure S2B).

We first compared GEM structure between primary tumors and matched normal tissues, followed by FBA of individual tumor models. We hypothesized that many tumors would have high metabolic demands to support their rapid proliferation. Accordingly, we used parsimonious FBA (pFBA), which first maximizes biomass production and fixes this while subsequently minimizing total intracellular flux. To generate more physiologically relevant fluxes, we used a radial diffusion model that utilizes relative concentrations and diffusion coefficients of metabolites in the blood to constrain metabolite uptake^21^ (Figure S2). Furthermore, we enzyme-constrained our models using the GECKO 3 pipeline^22^ to incorporate enzyme abundance and catalytic capacity, thereby limiting biologically unrealistic flux distributions that can arise from stoichiometric constraints alone. Using these flux values, we then examined the flux landscape across TCGA to investigate tissue-specific flux distributions, transcription factor (TF)-metabolic flux correlations, and metabolic flux differences between tumors harboring specific alterations in prominent driver pathways. Lastly, we applied machine learning approaches using metabolic flux values in multi-omics classifiers to predict cancer patient survival.

### Tissue-specific and conserved metabolic rewiring in cancer: Insights from GEMs

Unsupervised learning was used to explore the landscape of GEMs generated across various tumors and matched normal tissues. Previous pan-cancer analyses have demonstrated that tumor heterogeneity varies considerably across tissue types, motivating us to determine whether similar differences exist for metabolic network organization.^23^ As expected, patient samples clustered more closely according to their tissue of origin, including the matched normal samples (Figures 1A and S3A). We further characterized the structure of the generated GEMs by quantifying the number of genes, metabolites, and reactions retained across patient-specific models (Figures S3B–S3D). Consistent with tissue-specific differences in metabolic network organization, kidney tumors (KIRC, KIRP, and KICH) contained larger metabolic networks on average, whereas adrenal (ACC) and skin (SKCM) tumors displayed fewer retained genes, metabolites, and reactions. We also calculated inter-tumor heterogeneity (Figure S4A) and distances between tumors and their matched normal tissues (Figure 1B), observing that more closely related tissues displayed similar patterns of heterogeneity. For example, colon (COAD) and rectal (READ) tumors displayed lower levels of heterogeneity compared with adrenal tumors (ACC and PCPG), consistent with the diverse molecular phenotypes previously described in these tumors.^24^

**Figure 1:**
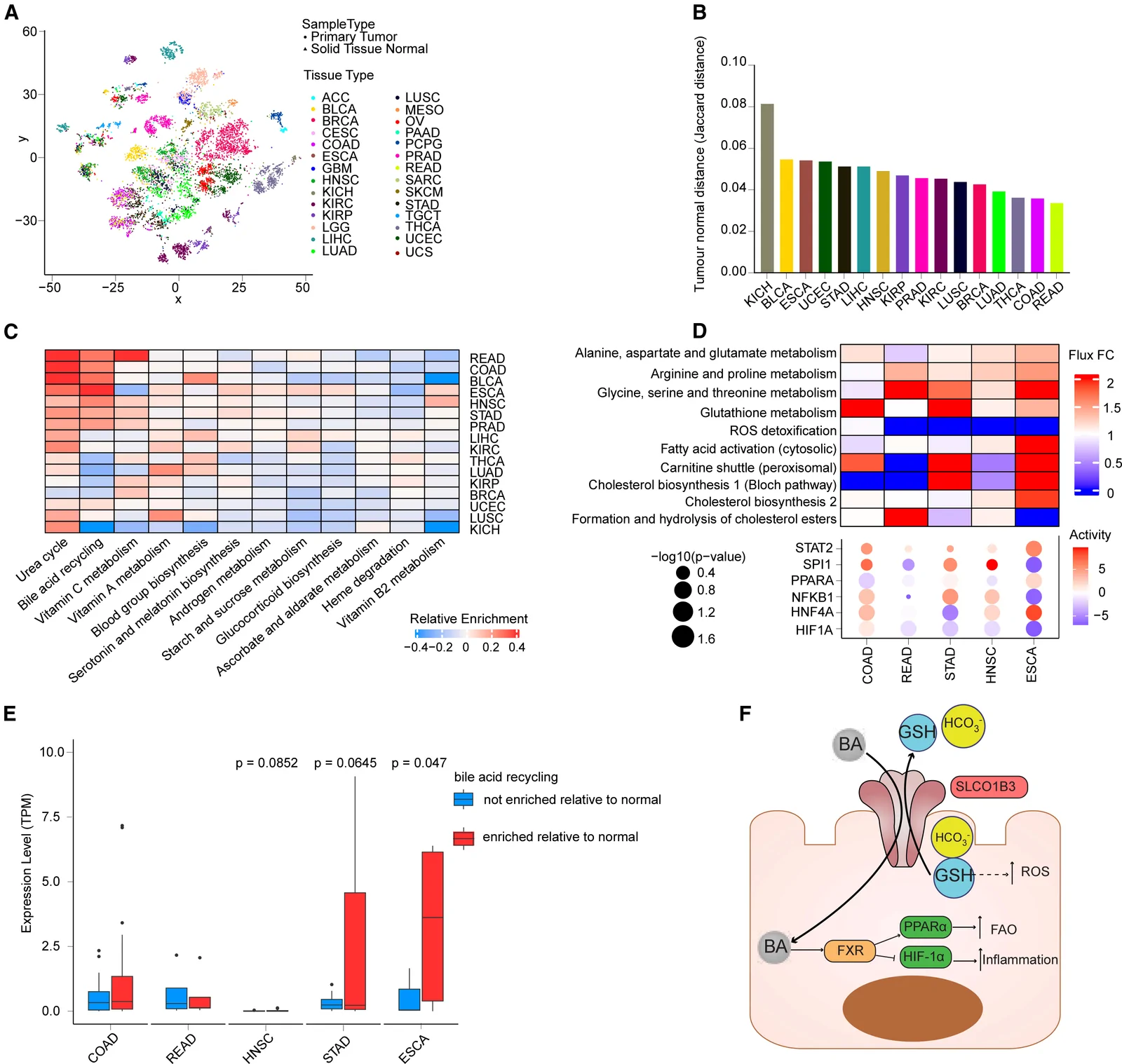
Bile acid recycling remodels redox homeostasis and drives tissue-specific lipid metabolic adaptation in gastrointestinal tumors (A) t-SNE visualization of all samples based on the Jaccard distance of metabolic reaction presence. (B) Average Jaccard distance between tumor and matched normal GEMs by tissue type. (C) Heatmap of metabolic subsystems differing between tumors and matched normal samples. Red and blue indicate enrichment in tumors and matched normal samples, respectively. (D) Differences in fluxes and transcription factor activity in GI tumors with and without increased bile acid recycling. Red and blue indicate higher and lower activity, respectively. (E) FXR expression across GI tumors. (F) Proposed mechanism linking bile acids to metabolic and signaling changes. *p* values in (D) were computed using two-sided *t* tests of TF activity coefficients and in (E) using one-way ANOVA with Tukey’s post hoc test. Analyses in (B) and (C) included 16 tissue types with ≥10 matched normal samples.

Of note, a small set of samples clustered together independently of their tissue of origin (Figures S4B and S4C). When comparing models from these “mixed” clusters with samples that clustered independently, we observed enrichment of specific metabolic pathways including the urea cycle, bile acid recycling, and vitamin B2 metabolism, among others (Figure S4D). Upon examining differences in metabolic sub-systems between tumors and their matched normal samples, we observed that several tumors across multiple tissue types shifted metabolism toward reactions in specific subsystems, most notably in the urea cycle and bile-acid recycling (Figure 1C). Urea cycle dysregulation has previously been reported in multiple tumor types to increase nitrogen utilization for *de novo* pyrimidine synthesis,^25^ providing internal validation for our results.

We subsequently investigated whether the pan-cancer flux atlas could distinguish shared metabolic programs across tumor types from tissue-specific adaptations. Among pathways recurrently altered across gastrointestinal tumors, bile acid recycling represented a unique opportunity to investigate how a common metabolic exposure, previously characterized in individual tissues,^26^ can drive divergent metabolic phenotypes depending on tissue context. Therefore, we used FBA to compare metabolic flux in tumors that upregulated BA transporters with those that did not, relative to their matched normal tissues. We restricted this analysis to GI tumor types with ≥10 normal samples and heterogeneous BA recycling, PAAD was excluded because of insufficient normals and LIHC because BA recycling was broadly elevated.

Our analysis revealed that certain metabolic pathways, such as reactive oxygen species (ROS) detoxification pathways, were downregulated in many GI tumors that upregulated BA recycling (Figure 1D). BAs have previously been shown to increase ROS levels, leading to subsequent resistance to apoptosis.^27^ The specific BA transporters, such as SLC1OB3, that were upregulated in these tumors are antiporter exchangers that exchange BAs for glutathione (GSH) and bicarbonate (HCO3-) molecules. A consequent intracellular reduction in GSH could explain the decreased ROS buffering capacity and observed phenotypes. Our FBA analysis demonstrated increased flux through GSH export reactions in tumors with elevated bile acid recycling (Figure S5A), without a corresponding increase in cytoplasmic GSH biosynthesis (Figure S5B). To investigate the functional contribution of SLC1OB3 to GSH metabolism, we performed small interfering RNA (siRNA)-mediated knockdown of SLC1OB3 (Figure S5C) followed by stable isotope tracing using L-cystine (3,3′-^13^C_2_). Our experiments demonstrated that bile acid treatment decreased intracellular labeled GSH abundance (Figure S5D) while increasing extracellular labeled GSH accumulation (Figures S5E and S5F). Knockdown of SLCO1B3 partially restored intracellular GSH levels, suggesting that bile acid transporter activity contributes to altered GSH compartmentalization (Figures S5D–S5F). Functionally, bile acid exposure decreased the GSH/GSSG ratio (Figure S5G) and increased intracellular ROS levels (Figure S5H) and these effects that were partially rescued following SLCO1B3 depletion. These findings suggest that bile acid transport can influence redox homeostasis through modulation of GSH availability, although additional transporter dependent and independent mechanisms are likely to contribute.

Given the known role of bile acids as regulators of lipid metabolism, we next investigated whether increased bile acid recycling was associated with broader alterations in lipid metabolic flux programs. Our FBA analysis identified tissue-specific differences in lipid-associated pathways, including fatty acid (FA) and cholesterol metabolism, between tumors with and without increased bile acid recycling (Figure 1D). Specifically, esophageal (ESCA) and stomach (STAD) cancers displayed increased lipid metabolic fluxes, including increased FA oxidation (FAO), whereas colorectal (COAD), rectal (READ), and head and neck (HNSC) tumors did not. Importantly, functional validation using Seahorse analysis demonstrated that bile acid exposure increased FAO selectively in esophageal and stomach cancer models, but not in colorectal or head and neck models (Figures S5I–S5L), supporting our atlas-derived prediction that shared bile acid exposure can induce distinct tissue-specific metabolic adaptations.

We next investigated potential mechanisms that could explain the tissue-specific metabolic differences observed between BA recycling-high tumors. To identify mechanisms underlying these differences, we first compared mutation frequencies between tumors with and without increased BA recycling but found no consistent differences. We therefore investigated bile acid-responsive transcriptional programs and examined expression of the nuclear receptor FXR, a key regulator of bile acid and lipid metabolism (Figure 1E).^28^ Within each tumor type, we compared FXR expression between tumors with enriched BA recycling and those without increased BA recycling. We observed significantly increased FXR expression specifically in BA recycling-high ESCA and STAD tumors, whereas FXR expression was not increased in BA recycling-high COAD, READ, and HNSC tumors. FXR has been shown to upregulate PPAR-α and downregulate HIF1-α,^29^ providing a potential link between bile acid signaling, redox, and lipid metabolism.^30^ Consistent with this, BA-high ESCA tumors showed increased PPAR-α activity, whereas ESCA and STAD tumors showed reduced HIF1-α activity (Figure 1D). While both ESCA and STAD tumors with increased bile acid recycling displayed alterations in lipid metabolic flux, increased *PPARA* expression was predominantly observed in ESCA tumors (Figure S6A). Consistent with this, several lipid metabolic enzymes identified through FBA contained promoter-associated PPAR-α binding motifs, suggesting that PPAR-α transcriptional activity may directly contribute to lipid metabolic rewiring in ESCA (Figure S6B). Conversely, the absence of increased *PPARA* expression or activity in STAD suggests that additional tissue-specific regulatory mechanisms may contribute to the observed lipid metabolic phenotypes.

These findings suggest that tissue-specific responses to bile acid recycling may be partially mediated by distinct transcriptional programs. In ESCA tumors, increased PPAR-α expression and activity were associated with enhanced lipid metabolic fluxes, including FAO, while promoter motif analysis demonstrated enrichment of PPAR-α binding site in several enzymes identified by FBA. Together, this supports a model whereby bile acid-associated activation of the FXR-PPAR-α regulatory axis contributes to lipid metabolic rewiring in a subset of ESCA tumors (Figure 1F). In contrast, although STAD tumors also displayed alterations in lipid metabolism, the absence of increased PPAR-α expression suggests that additional regulatory mechanisms may contribute to these metabolic adaptations. While further functional studies are required to establish direct therapeutic dependencies, these results highlight FXR-associated signaling pathways as potential regulators of metabolic rewiring in specific gastrointestinal tumor contexts. Taken together, these findings demonstrate that while tumor metabolic landscapes are largely tissue specific, certain pathways such as the urea cycle and bile acid recycling, are commonly altered across cancers, with downstream metabolic consequences shaped by distinct tissue-dependent regulatory programs.

### Identification of tumor-specific metabolic dependencies and alterations using GEMs

GEM structure can additionally reveal how tumors alter their underlying metabolic capabilities during tumor development. Using the same tumor and matched normal GEMs, we therefore examined metabolic functions that were differentially retained or utilized during tumorigenesis, allowing us to identify metabolic programs selectively altered across tumor contexts. We observed that many tumor types specifically lost the ability to produce urea from glutamine or degrade ornithine. This is likely because these tumors recycle nitrogen to synthesize pyrimidines, corroborating previous results (Figure 2A).^25^

**Figure 2:**
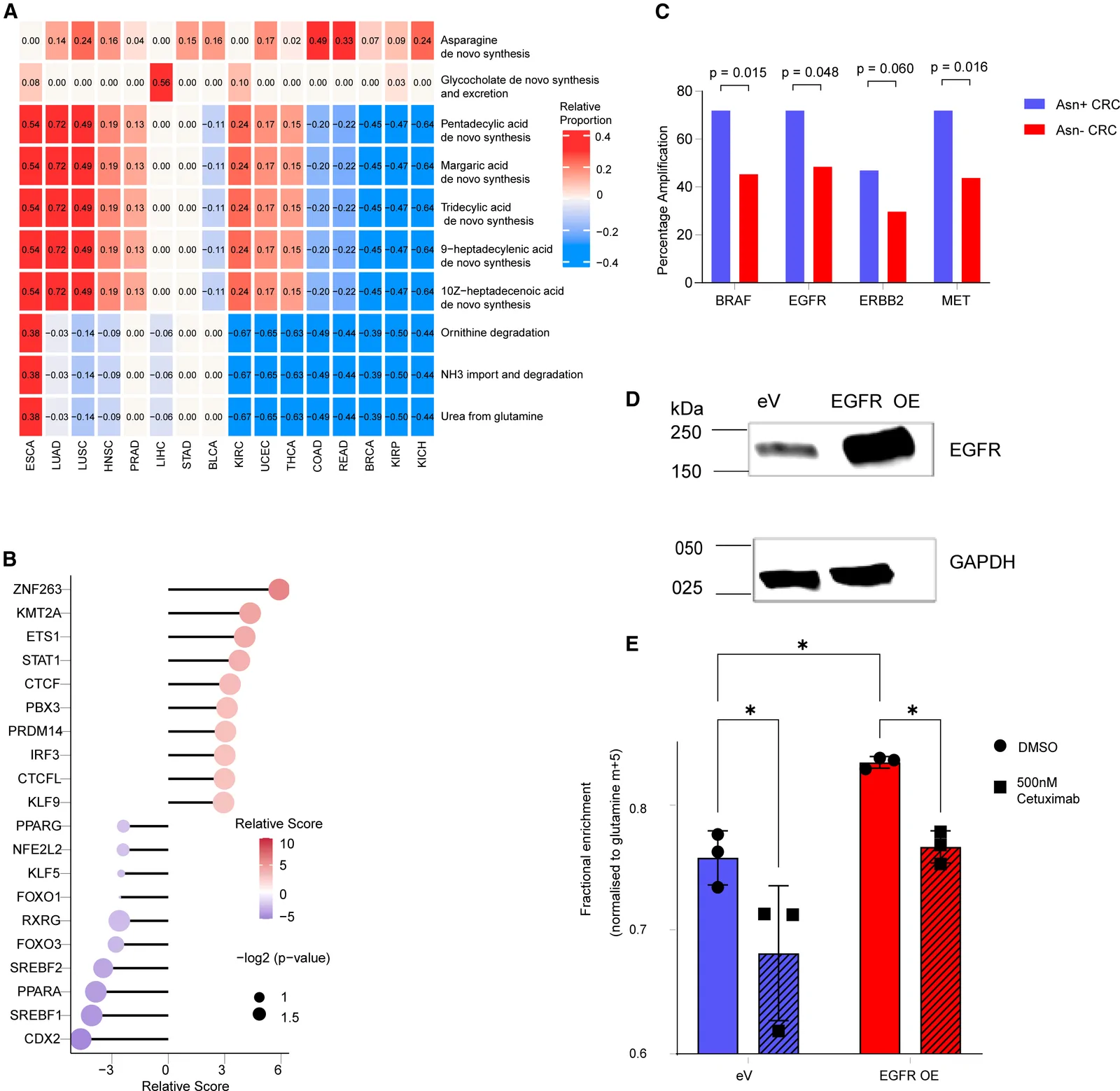
Colorectal tumors selectively upregulate *de novo* asparagine synthesis through distinct transcriptional programs (A) Heatmap of metabolic functions enriched or depleted in tumor versus matched normal GEMs. Values represent the proportion of tumors showing enrichment or depletion (−1 to 1). (B) Differences in transcription factor activity between ASNS-high and ASNS-low colorectal tumors in the validation cohort. (C) Frequency of copy number gains or amplifications in RTK-RAS genes in the colorectal validation cohort. (D) Western blot demonstrating EGFR overexpression in Caco-2 cells. (E) Fractional enrichment of asparagine from amide-15N-labeled glutamine in Caco-2 cells, comparing control and EGFR-overexpressing lines treated with or without 500 nM cetuximab. Data in (E) represent mean values ± SEM of *n* = 3 biological replicates. ns, not significant; ^∗^*p* < 0.05; ^∗∗^*p* < 0.01; ^∗∗∗^*p* < 0.001; *p* values in (B) were computed using two-sided *t* tests on TF activity coefficients, in (C) using Fisher’s exact test, and in (E) using two-way ANOVA with Tukey’s post hoc test. Analyses in (A) were carried out across 16 tissue types with at least 10 matched normal samples.

We also observed that approximately 50% of COAD and 33% of READ tumors upregulated *de novo* asparagine synthesis through ASNS relative to their matched normal tissue, indicating an increased reliance on asparagine. To extend our findings, we examined colorectal cancer (CRC) tumors in an additional cohort of 120 matched tumor-normal pairs.^31^ Here, we observed that 34% of tumors upregulated *de novo* asparagine synthesis relative to their normal counterparts, further corroborating our initial results.

To investigate why some CRC tumors upregulated ASNS, we compared TF activity, mutation frequencies, and copy-number alterations between ASNS-high and ASNS-low tumors. In TCGA, we observed increased activity of specific TFs, including ZNF263, PRDM14, CTCFL, and KLF9 (Figure S7A). These transcriptional changes were also observed in an independent CRC validation cohort containing 120 matched tumor-normal pairs (Figure 2B). We also detected increased amplification of genes involved in RTK/RAS signaling, including *EGFR*, *ERBB2*, *MET*, and *BRAF* (Figures 2C and S7B). Interestingly, ZNF263 showed the largest increase in activity across both cohorts and has been reported to be activated downstream of EGFR.^32^

To establish whether a relationship existed between asparagine synthesis and EGFR levels, we overexpressed EGFR in Caco-2 cells, a CRC line with no genetic alterations in proteins involved in RTK-RAS signaling (Figure 2D). We found that EGFR overexpression was sufficient to increase labeling from the amide group of glutamine into asparagine (Figure 2E), and this effect could be partially rescued by treatment with cetuximab, an EGFR inhibitor. While KRAS mutations have previously been shown to lead to ASNS upregulation in CRC tumors,^33^ our results suggest that increased *de novo* asparagine biosynthesis could also be driven by EGFR overexpression, highlighting potentially novel genotype-metabolic pathway relationships.

We also used GEMs to perform gene essentiality analyses and uncover metabolic vulnerabilities. Using matched normal samples allowed us to highlight specific metabolic vulnerabilities that would selectively inhibit tumor growth while sparing surrounding non-malignant tissue (Figure 3A). These included genes involved in core metabolic processes, such as nucleotide biosynthesis, peroxisomal FAO, and mitochondrial metabolism. Given the large number of predicted dependencies identified across tumor contexts, we focused subsequent analyses on genes predicted to represent tumor-selective vulnerabilities compared to matched normal tissues. Because GEM-based essentiality predictions can vary according to reconstruction methods, objective functions, and biological context, we treated them as candidate vulnerabilities requiring experimental validation. We therefore sought to determine whether the tumor-specific dependencies identified from our models corresponded to experimentally supported vulnerabilities.

**Figure 3:**
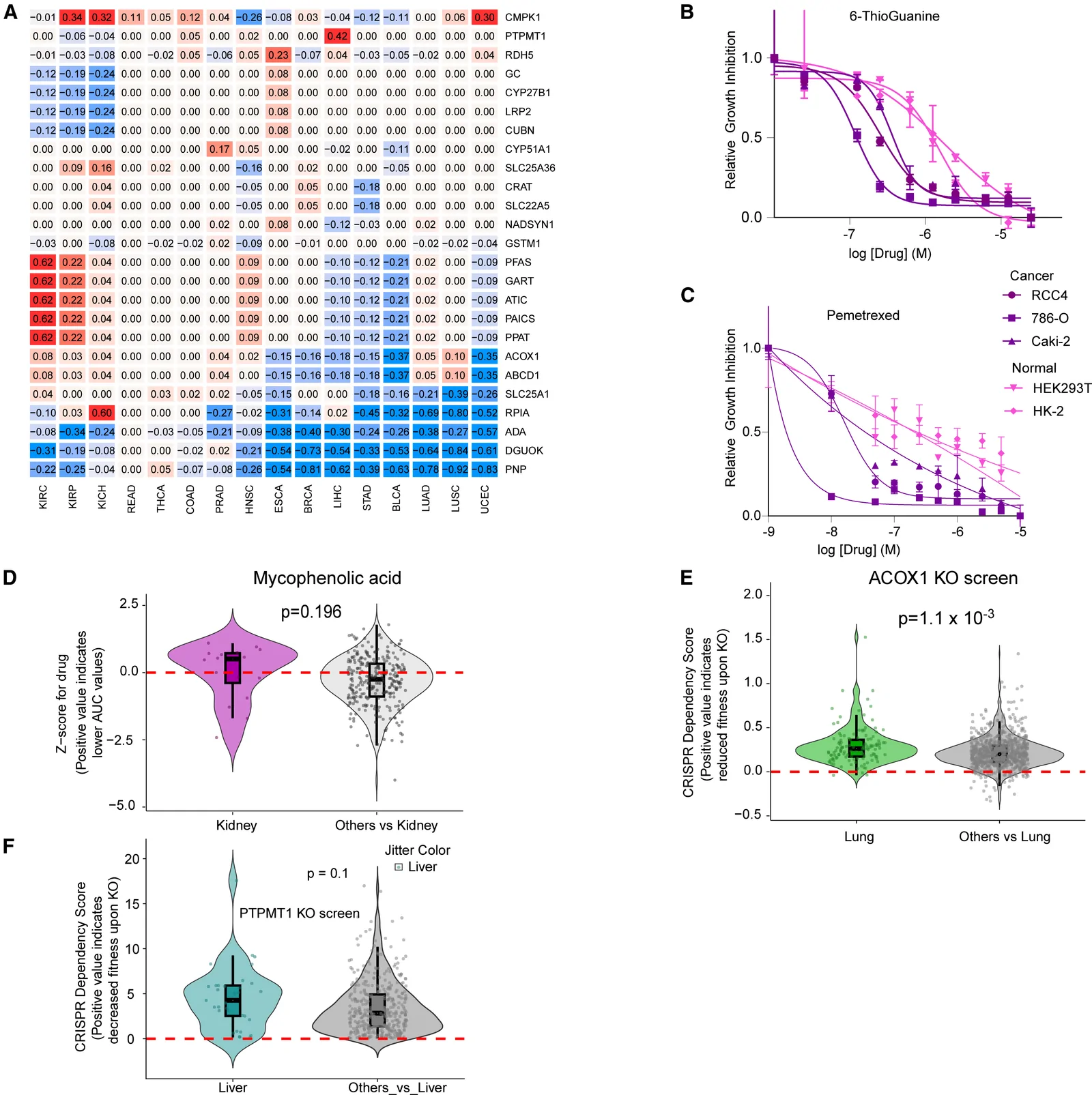
Gene essentiality analysis identifies tumor-selective metabolic dependencies validated across experimental and public datasets (A) Heatmap of essential genes enriched or depleted in tumor versus matched normal GEMs. Values represent the proportion of tumors showing decreased or increased dependency (−1 to 1). (B) Drug-response curves showing susceptibility to 6-thioguanine. (C) Drug-response curves showing susceptibility to pemetrexed in selected renal cancer and untransformed cell lines. (D) Genomics of drug sensitivity in cancer project (GDSC) scores for mycophenolic acid (MPA) in renal versus other cancer cell lines. (E) CRISPR dependency scores for ACOX1 in lung versus other cancer cell lines. (F) CRISPR dependency scores for PTPMT1 in liver versus other cancer cell lines. *p* values in (D)–(F) were calculated using two-way ANOVA with Tukey’s post hoc test. Analyses in (A) were carried out across 16 tissue types with at least 10 matched normal samples.

We subsequently experimentally validated our strongest context-specific dependencies, including the increased dependency of renal cancer cell lines on *de novo* purine synthesis (PFAS, GART, ATIC, PAICS, and PPAT) compared with untransformed renal cell lines (Figures 3B and 3C). Most drugs showed no strong cancer-versus-normal difference across other tissue origins, such as prostate or breast (Figures S8A–S8F). Renal cell lines also showed enhanced sensitivity to multiple purine synthesis inhibitors compared to cancer cell lines of other tissue origins. Conversely, untransformed renal cells were slightly more sensitive to forodesine, whereas renal cancer cells were not, consistent with a greater reliance on *de novo* synthesis than purine salvage. To further validate our results, we compared them with clustered regularly interspaced short palindromic repeats (CRISPR) knockout (KO) dependency scores for cancer cell lines available through DepMap and drug screening scores available from the Genomics of Drug Sensitivity in Cancer (GDSC). We showed that renal cell lines displayed enhanced sensitivity to mycophenolic acid (MPA), a purine synthesis inhibitor (Figure 3D), that lung tumors exhibited increased dependency on ACOX1 (Figure 3E), and that liver tumors showed increased dependency on PTPMT1 (Figure 3F). Although some comparisons did not reach statistical significance because limited sample numbers constrained statistical power, the direction of effect was consistent with our model predictions. *PTPMT1* dependency in liver cancer has also been reported previously.^34^^,^^35^ Collectively, these results suggest that while tumor metabolism is largely tissue specific, distinct vulnerabilities emerge from tissue- or genotype-specific metabolic pathway alterations, with metabolic flux analysis providing crucial insights about potential therapeutic targets.

### Pan-cancer fluxomic landscape reveals tissue-specific metabolic reprogramming and adaptation to nutrient stress

Previously constructed GEMs for tumor patients were subjected to FBA to interrogate the pan-cancer fluxomic landscape. To generate physiologically relevant flux distributions, we performed pFBA, which identifies solutions that maximize biomass production while minimizing unnecessary intracellular metabolic fluxes. The radial diffusion model allowed us to investigate metabolic flux under nutrient-replete conditions, where enzymatic constraints were the limiting factor for growth, and under nutrient-deprived conditions (Figure S2A). Under nutrient-replete conditions, ∼10% of tumors showed limited maximum growth potential, primarily from adrenal, renal, and melanoma lineages (Figures S9A–S9D). These tumors downregulated most growth-associated pathways while increasing tryptophan, arachidonic acid, and cholesterol metabolism (Figure S9E). Among tumors capable of high growth, tissue-specific flux patterns recapitulated known biology (Figure 4A): PAAD, STAD, and ESCA showed high glycolytic and glutamine flux (Figure S11A),^36^ prostate tumors showed high lipid metabolism and FAO (Figure S11B),^37^ and thyroid tumors showed high oxidative phosphorylation and pyruvate metabolism.^38^ CRC tumors showed both high glycolysis and FAO, consistent with metabolic plasticity. Nutrient uptake patterns similarly reflected tissue-specific metabolism (Figure S10A).

**Figure 4:**
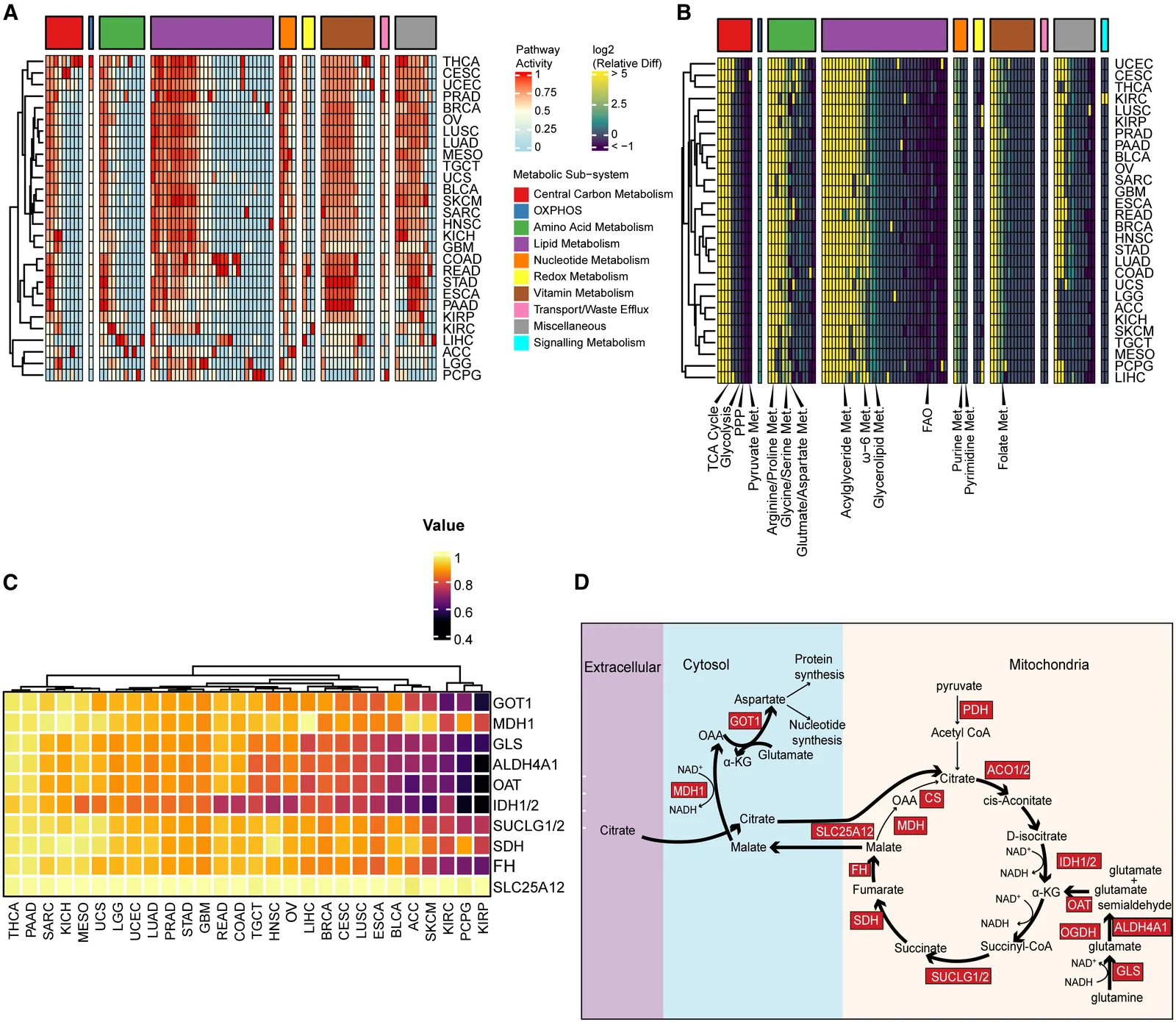
Nutrient limitation drives conserved metabolic adaptation while preserving tissue-specific metabolic states (A) Relative metabolic pathway fluxes averaged by tissue type and scaled from 0 to 1. (B) Changes in pathway flux under nutrient deprivation relative to nutrient-replete conditions, normalized to biomass. (C) Proportion of tumors upregulating reactions involved in glutamine entry into the TCA cycle. Enzymes catalyzing each reaction are indicated. (D) Schematic highlighting glutamine flux into the TCA cycle and aspartate synthesis under nutrient deprivation. NB: arrow thickness does not represent absolute FBA flux.

While nutrient limitation has been extensively studied in individual tumor models, whether cancers converge on common metabolic adaptations or utilize tissue-specific strategies remains unclear. We therefore leveraged our pan-cancer flux atlas to systematically compare metabolic responses to nutrient deprivation across tumor types and identify adaptive mechanisms shaped by tissue context. Overall, the data revealed that tumors mostly shared coordinated changes in pathways to sustain their growth under these conditions (Figure 4B) and showed a common preferential uptake of certain nutrients under nutrient deprivation (Figure S10B). Most tumors upregulated triglyceride synthesis, which is important for lipid-droplet formation, and simultaneously downregulated FAO. Lipid droplets are synthesized under nutrient stress conditions in cancer to serve as energy reservoirs, protect against lipotoxicity, and support membrane synthesis, thereby enabling cancer cells to survive and adapt to metabolic stress.^39^ We also observed increased folate metabolism, which sustained purine synthesis even in the presence of lower fluxes through the pentose phosphate pathway.^40^ Changes in folate metabolism flux enabled the specific production of NAD+ in the cytoplasm and NADH in the mitochondria, which would sustain energy production by supporting glycolysis and oxidative phosphorylation, respectively (Figure S11C).

A significant change observed across multiple tumor types was an increase in TCA cycle flux under nutrient deprivation (Figure 4C). Reaction-level analysis showed increased glutamine uptake and GLS-mediated conversion to glutamate, indicating a greater glutamine contribution to TCA-cycle activity (Figures 4C and 4D). To better quantify carbon entry into mitochondrial α-ketoglutarate, we next examined the relative contribution of glutamine- and citrate-derived carbon. While glutamine represented the dominant source of α-ketoglutarate in most tumor types, the contribution from citrate varied considerably between tissues, with renal tumors exhibiting a greater citrate contribution than pancreatic tumors (Figure S11D). Glutamine-derived intermediates were predominantly routed through oxidative metabolism to generate NADH, while a predicted TCA cycle “shortcut” exchanged mitochondrial malate for cytosolic citrate.

Analysis of the underlying reaction fluxes indicated that mitochondrial citrate was supplied via two routes: canonical citrate synthesis from pyruvate within the mitochondria, and import of cytosolic citrate through the mitochondrial citrate-malate antiporter. Cytosolic citrate was predicted to originate primarily from extracellular citrate uptake. While citrate is commonly considered an intracellular TCA cycle intermediate, circulating citrate is present in plasma and extracellular citrate uptake through transporters such as SLC13A5 has been reported to contribute to cellular metabolism.^41^ Cytosolic malate was subsequently converted into oxaloacetate, which fueled aspartate synthesis via GOT1. Aspartate is a critical amino acid with a variety of functions, including protein and nucleotide synthesis, and when aspartate levels are reduced in low-nutrient microenvironments, tumor cells must synthesize their own.^42^ We then examined the proportion of tumors that upregulate GLS and GOT1 activity and demonstrated that specific tumor types show a preferential use of glutamine, most notably thyroid and pancreatic tumors, whereas a much smaller proportion of renal and adrenal tumors upregulated GLS (Figure 4C).

To validate these predictions, we performed separate U-[^13^C] glucose (Figure 5A) and U-[^13^C] glutamine (Figure 5B) labeling experiments in three renal and pancreatic cancer cell lines under nutrient deprived and hypoxic conditions. These experiments showed that, under nutrient-deprived conditions, pancreatic cell lines depended more on oxidative glutamine flux into the TCA cycle, which fed into aspartate synthesis, as shown by enhanced m+4 labeling of succinate, fumarate, malate, and aspartate (Figure 5C). However, we did not observe differences in reductive glutamine flux into the TCA cycle between pancreatic and renal cancer cell lines. Meanwhile, renal cancer cell lines showed enhanced labeling of TCA cycle intermediates and aspartate from glucose, as shown by enhanced m+2 fractions of succinate, fumarate, malate, and aspartate (Figure 5D). In summary, these results suggest that our FBA approach was able to highlight metabolic flux patterns unique to specific tissues and elucidate how tumors adapt to metabolic stress induced by nutrient deprivation.

**Figure 5:**
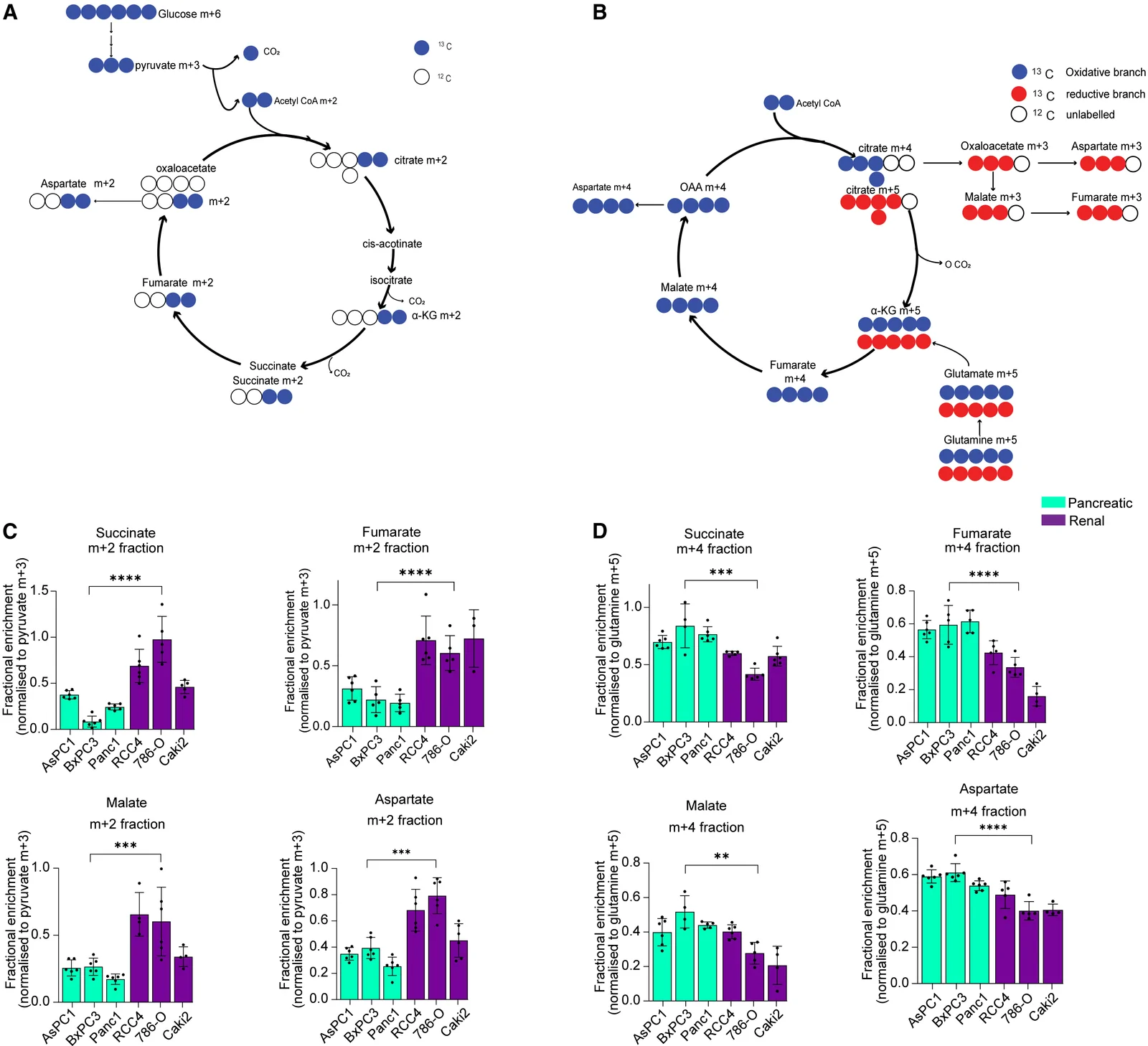
Stable isotope tracing validates tissue-specific nutrient adaptation predicted by metabolic flux modeling (A) Dominant U-[^13^C] glucose labeling pattern through the TCA cycle and aspartate synthesis. (B) Oxidative and reductive U-[^13^C] glutamine labeling patterns through the TCA cycle and aspartate synthesis. (C) Bar graphs showing various m+2 labeled fractions of TCA cycle intermediates and aspartate from glucose in pancreatic and renal cancer cell lines. (D) Bar graphs showing m+4 labeled fractions of TCA cycle intermediates and aspartate from glutamine in pancreatic and renal cancer cell lines. Data in (C) and (D) represent mean values ± SEM of *n* = 6 biological replicates. ns, not significant; ^∗^*p* < 0.05; ^∗∗^*p* < 0.01; ^∗∗∗^*p* < 0.001; *p* values in (C) and (D) were calculated using two-way ANOVA with Tukey’s post hoc test.

### TFs and driver alterations shape diverse metabolic landscapes in cancer

A fundamental question in cancer metabolism is which drivers control metabolic fluxes and whether different tissues share regulatory mechanisms. To address this, we computed TF activity in each tumor sample^43^ and correlated this with pathway metabolic fluxes. We focused on the top five tumor types that exhibited high fluxes in glycolysis and lipid metabolism (Figure 6A). Interestingly, we observed that glycolysis-associated TFs were largely shared across tissues, whereas lipid-associated TFs were more tissue specific. For tumors with high rates of glycolysis, we observed strong correlations with various TFs belonging to the hepatocyte nuclear factor (HNF) family and with CDX2, which regulates the development of the GI tract.^44^ HNF genes have established roles in liver development but have been increasingly recognized as underlying tumor metabolic reprogramming.^45^ Paradoxically, we also observed strong correlations between PPAR-α activity and glycolytic flux in GI tumors. PPAR-α is an important TF involved in upregulating FAO, and some studies suggest that it can suppress glycolysis.^46^ Given that several GI tumor types displayed high glycolytic flux (Figure S11A), we investigated whether PPAR-α activity was associated with additional metabolic differences. This revealed that GI tumors with higher PPAR-α activity showed increased glycolytic (Figure 6B) and FAO (Figure 6C) fluxes, suggesting that PPAR-α activity is associated with a subset of GI tumors exhibiting coordinated glycolytic and lipid-oxidative metabolism. GI tumors are often subject to widely differing nutrient availabilities,^47^ suggesting that some exhibit high metabolic plasticity, which could be coordinated by TFs such as PPAR-α.

**Figure 6:**
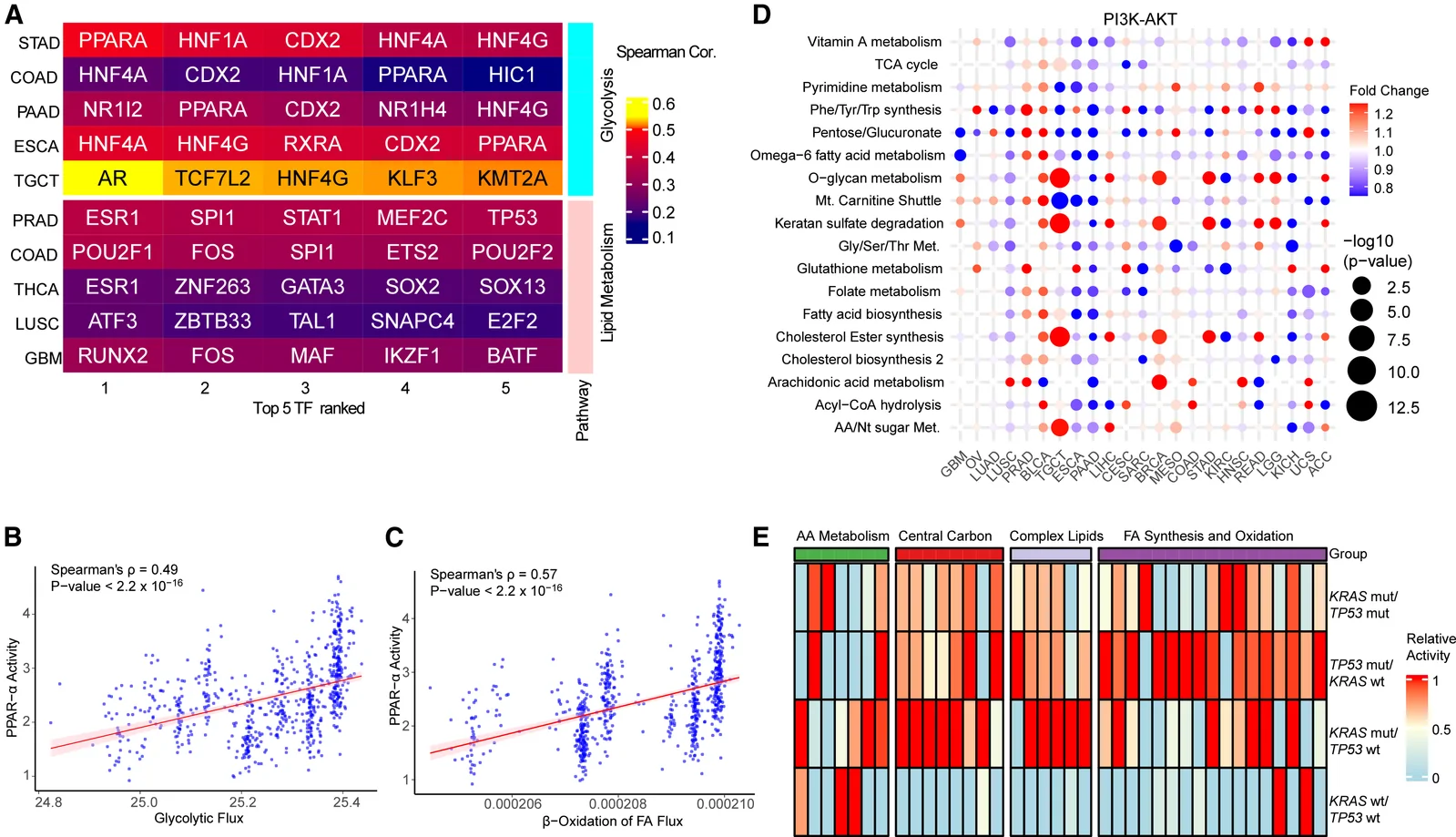
Distinct transcription factor networks and oncogenic pathways shape tissue-specific metabolic phenotypes (A) Top five transcription factors correlated with glycolytic and lipid metabolic flux in tissues with the highest pathway flux. (B) PPAR-α activity versus glycolytic flux in STAD, COAD, PAAD, and ESCA. (C) PPAR-α activity versus fatty acid β-oxidation flux in STAD, COAD, PAAD, and ESCA. (D) Flux changes associated with PI3K-AKT pathway alterations. (E) Heatmap depicting relative metabolic fluxes in lung tumors harboring mutations in both *TP53* and *KRAS*, *TP53* only, *KRAS* only, or wild type for both genes. *p* values in (B) and (C) were calculated from Spearman correlations between flux and TF activity and in (D) using one-way ANOVA, with correction for multiple hypothesis testing using the Benjamini-Hochberg correction.

We then turned our attention to known cancer driver pathways and asked whether the same alterations in different tissue types showed common metabolic patterns. Using pathway classifications from Sanchez-Vega et al.,^48^ we analyzed flux differences across tissues by comparing tumors with and without specific pathway alterations (Figures S12, S13 and S14). We recapitulated reported associations between PI3K-AKT and arachidonic acid metabolism in breast cancer^49^ (Figure 6D), cell cycle alterations and nucleotide synthesis (Figure S13E)^50^ and HIPPO signaling and cholesterol metabolism (Figure S14A).^51^ However, many of these associations were not reflected across all tissue types. We also observed that certain tissue types showed global metabolic alterations unique to specific pathway alterations. This is exemplified by MYC, which is known to be an important driver of metabolism.^52^ Some tissue types, including BLCA, ESCA, STAD, and PAAD all showed upregulations in multiple metabolic pathways in MYC-altered tumors, but other tissue types did not (Figure S14B). This indicates that the effect of pathway drivers on tumor metabolism is highly tissue-type dependent.

Using the example of *KRAS* and *TP53* alterations in lung tumors, we further investigated whether alterations in multiple pathways had synergistic or antagonistic effects on metabolism (Figure 6E). Comparing tumors harboring *KRAS* mutations, *TP53* mutations, or concurrent *KRAS/TP53* mutations revealed that double-mutant tumors exhibited metabolic profiles that were not simply explained by the individual alterations alone, suggesting that interactions between oncogenic drivers can shape context-specific metabolic states. In metabolic pathways involving amino acids, central carbon metabolites, and complex lipids, double mutant (MUT) tumors more strongly resembled *KRAS* WT (wild-type)/*TP53* MUT tumors. In contrast, for FA synthesis and oxidation, double MUT tumors instead resembled *KRAS* MUT/*TP53* MUT tumors (Figure 6E).

### Leveraging fluxomics to identify clinically relevant biomarkers

Because many circulating metabolites can be measured using minimally invasive approaches and therefore represent attractive biomarker candidates, we investigated whether fluxomic data could predict patient survival and identify novel biomarkers. To achieve this, we applied various machine learning algorithms to our fluxomics data, which included both pathway-level and individual reaction fluxes, to predict survival (Figure S15A). We found that Random Forest survival models performed best, yielding a Uno’s concordance-index (c-index) score of approximately 0.65. We also compared our models with those trained on gene mutation data, inferred TF activity, and clinical features as well as an ensemble model that combined predictive features from mutation, TF activity, clinical, and metabolic flux-based models (Figure 7A).

**Figure 7:**
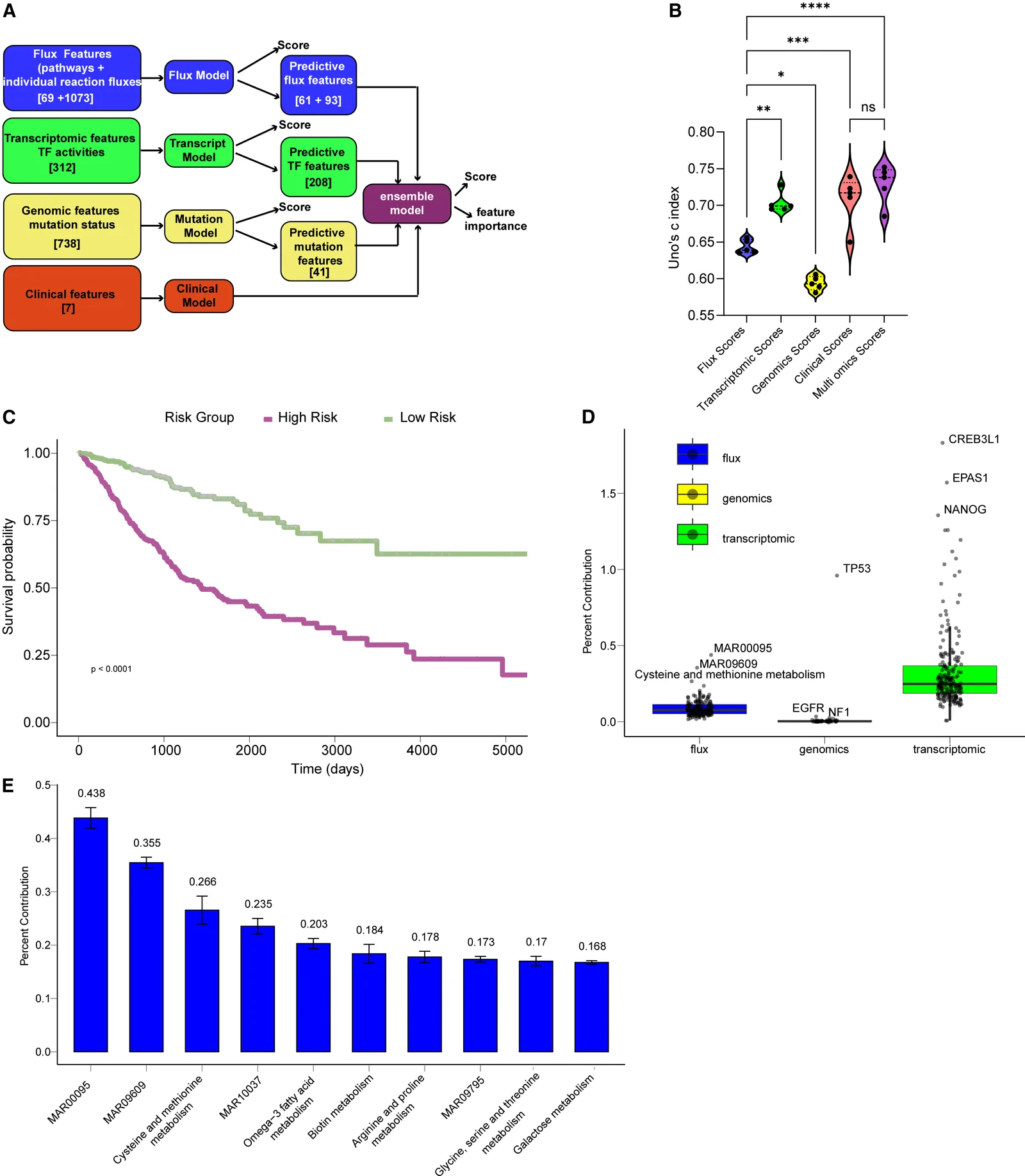
Metabolic flux identifies prognostic biomarkers for predicting patient survival (A) Machine-learning workflow for individual data modalities and the integrated ensemble model. (B) Best model performance across data modalities and the ensemble model using 5-fold cross-validation stratified by tissue type. (C) Kaplan-Meier analysis of flux-based risk scores in the test set. The highest and lowest 25% of predicted risk scores were classified as high- and low-risk, respectively. The *p* value in (C) was calculated using a log rank test. (D) Boxplots demonstrating feature importance for omics features in the ensemble model. For each data modality, the top three features are labeled. (E) Barplots of flux feature importance in the ensemble model. *p* values were computed using one-way ANOVA with Tukey’s post hoc test.

We found that models trained on fluxomic data performed better than those trained on gene mutation data but worse than those trained on transcriptomic or clinical data (Figure 7B). We reasoned that this could be due to a lack of patient-specific constraints, the context dependency of metabolism, and the inference of fluxes rather than direct measurement. Despite the relatively lower c-index values, models trained on flux data were able to correctly classify “high-risk” and “low-risk” patients in unseen test data based on survival (Figure 7C). Furthermore, flux features significantly contributed to the predictive power of the ensemble model (Figures 7D and S15B), suggesting that our fluxomic data have potential for predicting cancer patient survival.

We then examined the flux features that contributed most to the ensemble model’s predictions. We found that the top two features (MAR00095 and MAR09609) were reactions involved in biotin uptake (Figure 7E). Biotin is essential in cancer because of its role as a cofactor for carboxylase enzymes involved in FA synthesis, gluconeogenesis, and amino acid metabolism, which are crucial for rapidly proliferating cancer cells.^53^ While studies have underscored biotin’s role in cancer progression, relatively little work has established biotin as a biomarker. Our models suggest that more aggressive cancers exhibit greater biotin uptake and have a worse prognosis (Figure S15C), pointing to biotin as a potential novel biomarker for cancer diagnosis, alongside other relevant features identified by our models (Figures S15D–S15F). Overall, our models suggest that FBA-derived fluxomic data have potential for predicting cancer patient survival and can reveal overlooked biomarkers with clinical potential.

## Discussion

Building on the proven utility of GEMs^54^^,^^55^ we developed FluxAtlas, a pan-cancer resource of patient-specific metabolic flux states integrating transcriptomic, enzyme, and physiological nutrient constraints. Across >10,000 TCGA tumors, FluxAtlas identified conserved and tissue-specific metabolic programs, candidate dependencies, nutrient-stress adaptations, and prognostic features. By integrating fluxomic data generated from these models with information on genetic alterations, transcriptional regulation, metabolic dependencies, and experimental validation, our analysis demonstrates how genome-scale flux atlases can be leveraged not only to characterize metabolic differences but also to identify context-specific metabolic behaviors and vulnerabilities across cancers.

We first examined overall differences in metabolic rewiring between tumor tissues and their matched normal equivalents. We found that certain pathways, including urea and bile acid recycling, were commonly dysregulated across multiple tissue types. While BAs have been demonstrated to be potent carcinogens in several GI tumors, their exact downstream metabolic consequences in different cancer tissue types have not been fully established. Our data demonstrated that most GI tumors significantly downregulated ROS detoxification fluxes as they exchanged BAs for GSH. Increased ROS may also indicate that BA-recycling tumors could be more sensitive to radiotherapy because of reduced ROS buffering capacity^56^ and BAs could serve as effective biomarkers for patients who should receive radiotherapy, although this requires further investigation. We also predicted that esophageal and stomach cancers respond differently to BAs than other GI tumors. This differential response to BAs is underpinned by differences in the expression of the TF FXR, which mediates downstream increases in FAO. FXR is gaining increasing attention as a potential therapeutic target, and our investigation highlights that it could offer increased benefit for certain cancer types.

We also examined differences in metabolic function by comparing tumors with their matched normal tissues. Specifically, we identified upregulation of asparagine synthesis via ASNS in colorectal tumors. The importance of asparagine for tumor growth has been demonstrated by the clinical success of extracellular asparaginase in treating low-ASNS-expressing leukemia.^57^ However, tumors may not exhibit sensitivity to asparaginase treatment if they possess the ability to synthesize asparagine *de novo*. Our investigation showed that many CRC tumors upregulate ASNS, likely in association with increased RTK/RAS signaling. Interestingly, MAPK inhibition attenuates ATF4-mediated expression of ASNS in melanomas,^58^ and KRAS mutations have been implicated in upregulating ASNS expression in CRCs.^33^ This suggests that CRC tumors with RTK/RAS alterations may benefit from a combination of RTK/RAS and ASNS inhibition, representing a promising new clinical avenue for CRC treatment. Our study also revealed that the TF ZNF263 was most strongly associated with enhanced ASNS expression. ZNF263 activation has been reported downstream of EGFR signaling^59^ in lung tumors and glioblastomas,^32^ and CRC tumors with high ASNS expression exhibit higher EGFR amplification. Whether ZNF263 directly regulates ASNS remains to be determined. Matched tumor-normal GEMs also identified candidate tumor-selective dependencies, most prominently increased reliance on *de novo* purine synthesis in renal tumors, which was validated *in vitro*. Purine *de novo* biosynthesis is essential in tumors because it provides a continuous supply of nucleotides to sustain proliferation. As GEM-derived essentiality predictions depend on model reconstruction, objectives, and regulatory assumptions, they should be viewed as hypotheses requiring experimental validation.

We then turned our attention to examining flux across the pan-cancer metabolic landscape. By constraining nutrient uptake using a radial diffusion model, we were able to examine metabolic fluxes under nutrient-replete and nutrient-deprived conditions. However, it is important to recognize that pFBA represents one possible solution within the feasible metabolic solution space by maximizing biomass while minimizing intracellular fluxes. Flux sampling or alternative objectives may capture additional states, particularly in normal tissues, where proliferation is not the sole objective. Our nutrient-deprivation analysis nevertheless identified conserved adaptations, including increased folate metabolism and lipid storage, reduced FAO, and increased glutamine contribution to TCA-cycle activity and aspartate synthesis via a citrate-malate exchange in the mitochondria. However, given the diverse roles of citrate metabolism, including ACLY-dependent cytosolic citrate utilization and glutamine-dependent reductive carboxylation, this predicted flux route represents one possible metabolic configuration identified by pFBA rather than a unique solution. These findings suggest that tumors converge on common adaptive strategies under nutrient stress despite tissue-specific metabolic preferences.

Our study also correlated TF activities with metabolic flux, identifying key transcriptional regulators that may drive tumor metabolic reprogramming. Interestingly, our analysis demonstrated that specific TFs were strongly correlated with glycolytic flux across multiple tissue types, but for lipid metabolism, the correlations tended to be tissue specific. Our analysis highlighted the HNF family of TFs as highly correlated with glycolysis. The HNF family, particularly HNF4A, is abnormally expressed in multiple tissue types and has established roles in regulating genes involved in glucose homeostasis, lipid metabolism, and amino acid processing.^45^ However, their context-dependent functions, including both tumor promoting and tumor-suppressive effects, present challenges for therapeutic targeting.^45^ FBA may help identify context-specific strategies that minimize effects on normal tissue metabolism.

Additionally, we examined the impact of common pathway alterations on metabolic flux in a tissue-specific manner. While we recapitulated several previously reported associations, our analysis also revealed tissue-specific effects of oncogenic alterations on broader metabolic programs. This was particularly evident for MYC: in bladder, stomach, and esophageal cancers, MYC amplification was associated with increased flux across multiple metabolic pathways. We also extended our analysis to examine the effect of multiple drivers on lung cancer metabolism. Strikingly, we observed that tumors with mutations in both *TP53* and *KRAS* resembled their single MUT counterparts for select metabolic pathways. This observation indicates that context is crucial for interpreting a cancer driver’s effect on metabolism.

We concluded our study by investigating whether flux-derived metabolic features contained prognostic information associated with patient outcomes. Flux-based models showed modest prognostic performance, outperforming mutation-based models but not TF activity or clinical models. However, flux-based models were able to distinguish patients with different survival outcomes, suggesting that metabolic states inferred from FBA capture clinically relevant aspects of tumor biology. Given the pan-cancer nature of TCGA, these associations should be interpreted in the context of underlying cohort heterogeneity, including differences in tissue subtype, clinical management, and treatment regimens that cannot be fully accounted for using available clinical annotations. Furthermore, the modest predictive performance of the survival models likely reflects inherent limitations of the current modeling framework, including the fact that FBA fluxes are inferred from transcriptomic data and do not capture post-transcriptional regulation, enzyme abundance, metabolite availability, or temporal metabolic adaptation.

Nevertheless, the ability of flux-derived features to contribute prognostic information suggests that inferred metabolic states capture biologically meaningful aspects of tumor behavior. From our models, we identified metabolic features associated with patient prognosis, including metabolites such as biotin, highlighting potential avenues for future biomarker development. Previous studies have demonstrated the potential of integrating GEM modeling with machine learning to extract clinically and biologically meaningful metabolic features that predict specific therapeutic phenotypes, including radiotherapy response,^60^ although differences in modeling approaches and endpoints make direct comparison with our study challenging. Here, we offer complementary analyses by evaluating whether enzyme constrained, physiologically informed flux predictions capture prognostic metabolic states associated with overall patient survival.

Overall, our study provides a pan-cancer framework for investigating tumor metabolism through personalized flux modeling, enabling the identification of metabolic phenotypes, regulatory programs, and context-specific vulnerabilities. Future work incorporating additional clinical variables, longitudinal sampling, and tumor-specific objective functions will further refine these approaches and improve our ability to model the dynamic nature of cancer metabolism.

### Limitations of the study

Several limitations should be considered. First, pFBA identifies a single optimal flux distribution and therefore does not represent the full range of feasible metabolic states, alternative objective functions or flux-sampling approaches may reveal additional solutions. Second, patient-specific GEMs incompletely capture post-translational regulation, metabolite availability, tissue microenvironmental influences, and mutation-specific effects on enzyme activity. Third, the predicted metabolic dependencies require experimental validation. Finally, survival analyses may be affected by clinical and treatment-related variables that are incompletely annotated or inconsistently captured across TCGA cohorts. Future studies integrating longitudinal molecular, metabolic, and clinical data should enable more context-specific and dynamic modeling of cancer metabolism.

## Resource availability

### Lead contact

Requests for further information and resources should be directed to and will be fulfilled by the lead contact, George Poulogiannis (george.poulogiannis@icr.ac.uk).

### Materials availability

This study did not generate new unique reagents.

### Data and code availability

• All transcriptomics datasets used in our study are publicly available. Human-GEM model was accessed from Robinson et al*.*^19^ TCGA pan-cancer RNA-seq TPM, Raw Counts, and genomics data were accessed through the R package TCGABiolinks. Data from the colorectal validation cohort are available online: short somatic variant call, copy number variation (CNV), and structural variant (SV) data are available at the European Variation Archive^61^ under accession number PRJEB61514, and expression profiles at the ArrayExpress^62^ under accession number E-MTAB-12862. Metabolomics data generated in this investigation are available as Table S1.
• The code used to generate GEMs, perform gene essentiality, assessment of metabolic task and FBA, and the ML pipelines are all available through GitHub (https://github.com/manas-kohli/Pan_Cancer_FBA). Files used for analysis are also available on the repository.
• The code used for analysis in the Figures will be made available upon reasonable request. Any additional information required to reanalyze the data reported in this paper is available from the lead contact upon request.

## Acknowledgments

Work in the G.P. lab was supported by 10.13039/100014013UK Research and Innovation (MR/W012030/1 and MC_PC_MR/X013715/1). The authors wish to acknowledge the support of ICR’s Research Software Engineering group on building and maintaining the website associated with this study.

## Author contributions

M.K. performed and analyzed most experiments with contributions from A.T., M.K., and G.P. conceptualized the idea, designed the study, and wrote the manuscript. A.S. built the website associated with this study. G.P. supervised the study. All authors edited the manuscript.

## Declaration of interests

The authors declare no conflict of interest.

## STAR★Methods

### Key resources table

**Table:** 

REAGENT or RESOURCE	SOURCE	IDENTIFIER
**Antibodies**		

Rabbit monoclonal anti-EGF-Receptor	Cell Signaling Technology	Cat# 4267; RRID: AB_2864406
Rabbit monoclonal anti-GAPDH	Cell Signaling Technology	Cat# 2118; RRID: AB_561053
Mouse monoclonal anti-Rb (4H1)	Cell Signaling Technology	Cat# 9309; RRID: AB_823629

**Chemicals, peptides, and recombinant proteins**		

Hydrocortisone	Sigma-Aldrich	Cat# H-0888
Epidermal Growth Factor (EGF)	Peprotech	Cat# AF-100–15
Cholera toxin	Sigma-Aldrich	Cat# C-8052
Insulin	Sigma-Aldrich	Cat# I-1882
Streptomycin sulfate salt	Sigma-Aldrich	Cat# S9137
Benzylpenicillin Sodium	Britannia Pharmaceutical Limited	N/A
Methanol HPLC grade	Fisher Scientific	Cat# A456-212
Acetonitrile HPLC grade	Fisher Scientific	Cat# A955-212
H_2_O HPLC grade	Fisher Scientific	Cat# W/112/17
Ammonium acetate	Fisher Scientific	Cat# A/3446/50
InfinityLab Deactivator Additive	Agilent	Cat# 5191–4506

**Deposited data**		

Enzyme turnover numbers	BRENDA database	https://www.brenda-enzymes.org
FBA models of TCGA tumors	This paper	https://github.com/manas-kohli/Pan_Cancer_FBA
TCGA clinical data	The Cancer Genome Atlas; Synapse TCGA_Pancancer project	https://www.synapse.org/#!Synapse:syn300013/wiki/27406
TCGA genomic data	The Cancer Genome Atlas; GDC data portal	https://portal.gdc.cancer.gov
TCGA transcriptomic data	The Cancer Genome Atlas; (Rahman et al.^63^)	GEO: GSE62944

**Experimental models: cell lines**		

MCF10A	ATCC	Cat# CRL-10317
T47D	ATCC	Cat# HTB-133
MDAMB231	ATCC	Cat# CRM-HTB-26
Caco-2	ATCC	Cat# HTB-37
KM12	Accegen	Cat# ABC-TC5010
RCC4	Sigma-Aldrich	Cat# 03112702-1VL
786-O	ATCC	Cat# CRL-1932
Caki-2	ATCC	Cat# HTB-47
AsPC1	ATCC	Cat# CRL-1682
BxPC3	ATCC	Cat# CRL-1687
Panc1	ATCC	Cat# CRL-1469
PNT2	Sigma-Aldrich	Cat# 95012613-1VL
PC3	ATCC	Cat# CRL-1435
22RV1	ATCC	Cat# CRL-2505
DU145	ATCC	Cat# HTB-81
HK-2	Cytion	Cat# 305021

**Oligonucleotides**		

ACAGCAGAGTCAGCATCTTCAG	Sigma-Aldrich	SLC1OB3-Fwd
ATCACAAGCAAATTTCCAATTT	Sigma-Aldrich	SLC1OB3-Rev

**Software and algorithms**		

R statistical software (Version 4.4.2)	The R Project	https://www.r-project.org/
MATLAB (2021c, version 9.11)	Mathworks	https://www.mathworks.com/products/matlab.html
GraphPad Prism (version 10.6.1)	GraphPad	https://www.graphpad.com/scientific-software/prism/
Source data and original code	This paper	https://github.com/manas-kohli/Pan_Cancer_FBA
Optimization software	Gurobi 10.0.1	https://www.gurobi.com/
Python 3.9.6	Python	https://www.python.org/downloads/release/python-396/

### Experimental model and study participant details

#### Cell lines and culture conditions

Human cell lines used in this study included both non-malignant and cancer-derived models from diverse tissue origins. MCF10A is a non-tumorigenic mammary epithelial cell line derived from fibrocystic breast tissue and served as a normal breast model. T47D and MDA-MB-231 are breast cancer cell lines, representing luminal A (ER-positive, PR-positive, HER2-negative) and triple-negative subtypes, respectively. Caco-2 and KM12 are colorectal adenocarcinoma cell lines, while RCC4, 786-O, and Caki-2 are renal cell carcinoma lines derived from clear cell kidney tumors. Pancreatic ductal adenocarcinoma was represented by the AsPC-1, BxPC-3, and PANC-1 cell lines. PNT2 is a non-malignant prostate epithelial cell line. Prostate cancer models included PC-3, 22Rv1, and DU145, derived from advanced or metastatic disease. Kidney-derived non-malignant cell lines comprised HK-2, a proximal tubule epithelial cell line, and HEK293T, a human embryonic kidney cell line transformed with SV40 large T antigen. Gastrointestinal models included OE33, an esophageal cancer cell line; AGS, a stomach cancer cell line; CAL27, a head and neck cancer cell line; and HCT116, a colorectal cancer cell line.

Cell lines were purchased from the American Type Culture Collection (ATCC), Cytion, Sigma-Aldrich or Accegen. The respective media they were grown in can be found in Table S2. All media, with the exception of MCF10A, were supplemented with 10% Fetal Bovine Serum (FBS), and cells were cultured at 37°C in a 5% CO_2_ incubator. Cell lines were authenticated using the Human Cell Line Authentication service from Eurofins. Mycoplasma testing was also performed using the MycoplasmaCheck service from Eurofins.

MCF10A cells were cultured in DMEM/F-12 supplemented with 5% horse serum, 20 ng/mL epidermal growth factor (EGF), 100 ng/mL cholera toxin, 0.5 mg/mL hydrocortisone, 10 μg/mL insulin, 100 IU/mL penicillin and 100 μg/mL streptomycin (PEST). For dose-response experiments testing the effects of purine synthesis inhibition, 5% horse serum was replaced with 5% dialyzed FBS.

To evaluate metabolic labeling under nutrient deprivation, pancreatic and renal cancer cell lines were first adapted to stress conditions for two weeks. Cells were cultured in DMEM lacking glucose, glutamine, sodium pyruvate, and FBS and supplemented with 1 mM glucose, 0.2 mM glutamine, and 1% FBS. Cultures were maintained in a Whitley H35 Hypoxystation (Don Whitley Scientific) set to 1% O_2_, 5% CO_2_, and 94% N_2_ at 37°C and 80% humidity. During the adaptation period, most cells underwent cell death; only cells that survived and resumed proliferation were used for subsequent labeling experiments.

### Method details

#### Human metabolic models

Genome-scale metabolic models (GEM) were generated using Human1 as a reference. This model comprises 13,082 reactions and 8,378 metabolites, distributed across nine distinct compartments: the extracellular space, peroxisome, mitochondria, cytosol, lysosome, endoplasmic reticulum, Golgi apparatus, nucleus, and inner mitochondrial membrane.

#### Model generation using ftINIT

Personalized human metabolic models were then constructed using Human1 as the input model and RNAseq data from individual samples, together with the fast Task-driven Integrative Network Inference for Tissues (ftINIT) algorithm. ftINIT is an optimized implementation of the original tINIT approach for efficient generation of context-specific GEMs. A transcripts-per-million (TPM) threshold of 1 was applied. ftINIT was selected for context-specific model reconstruction because it enables the integration of transcriptomic data while preserving the ability of reconstructed models to perform essential metabolic functions. Unlike approaches based solely on removing reactions with low expression, ftINIT incorporates metabolic task constraints during reconstruction to ensure that the generated GEMs remain biologically functional. Additionally, the computational efficiency of ftINIT enabled scalable generation of patient-specific models across the entire TCGA cohort (tissue type abbreviations can be found in Table S3) while maintaining compatibility with the Human1 metabolic reconstruction. First, the reference metabolic model was converted into closed form by incorporating boundary metabolites. After reconstruction, the biological feasibility of each model was assessed by verifying its ability to perform essential metabolic functions common to all cell types. A set of 57 metabolic tasks necessary for cellular viability was used for this evaluation. MATLAB’s implementation of the tINIT algorithm was used for this step.

#### Gene essentiality and metabolic function analysis

Following model reconstruction, gene essentiality analysis was performed by systematically simulating single-gene knockouts and evaluating their impact on the metabolic network. Each metabolic gene was individually removed from the model, and FBA was performed to determine the impact on biomass production. Genes were classified as essential if their deletion completely abolished the ability of the model to produce biomass (biomass flux < 1e−6), representing metabolic genes predicted to be strictly required for maintaining cellular growth. This stringent cutoff was selected to identify complete loss-of-growth phenotypes rather than partial reductions in predicted proliferation.

Each model was also assessed for its ability to perform specific metabolic tasks using 256 pre-defined tasks. A model’s ability to perform a specific metabolic task depends on whether it contains the necessary inputs and reactions to enable the desired metabolic function (e.g., *de novo* asparagine synthesis). The complete list of metabolic tasks was obtained from the Human-GEM repository (metabolicTasks_Full.txt), which contains predefined metabolic functions used to evaluate the capability of a GEM to perform specific biological processes. Task completion represents the presence of a functional metabolic route capable of performing a defined biological process and was evaluated independently of the FBA-derived flux analyses. Relative proportions represent the fraction of tumor or normal GEMs within each cancer type capable of completing each metabolic task.

#### Enzyme-constrained models using GECKO

Constructed models were enzyme-constrained using the GECKO (Genome-scale model Enhanced with Enzymatic Constraints using Kinetic and Omics data) framework, as described previously.^22^ Enzyme constraints were incorporated by limiting reaction fluxes based on enzyme turnover numbers (*k*_*cat*_ values) and a predefined total protein pool. Total protein allocation was set as a global constraint, distributing available protein mass across enzymatic reactions.

#### Flux Balance Analysis (FBA)

A metabolic network can be represented by a stoichiometric matrix *S* of size *m*× *r*, where *m* and *r* denote the number of metabolites and reactions, respectively. Each entry *S*_*ij*_ corresponds to the stoichiometric coefficient of metabolite *i* in reaction *j*, with *S*_*ij*_ < 0 for reactants, *S*_*ij*_ > 0 for products, and *S*_*ij*_ = 0 if metabolite *i* is not involved.

The relationship between reaction fluxes (*r*, an 1 × *r* vector) and metabolite concentrations (*m*, an *m* × 1 vector) is given by:

Sv=0

FBA assumes a steady-state condition, meaning metabolite concentrations remain constant over time. Each reaction flux *v*_*j*_ is constrained within specified lower and upper bounds, which were defined by enzyme-constraining the model (see enzyme-constrained section):

$${v\;}_{\min,j}\leq v_{j}\leq {v\;}_{\max,j}$$

To determine feasible flux distributions, FBA formulates an optimization problem that maximizes an objective function f(v):

Maxf(v)

$$\text{subject}\;\text{to}\;Sv=0,\text{and}\;v_{\min,j}\leq v_{j}\leq v_{\max,j}$$

Solving this linear programming (LP) problem yields fmax, the optimal value of the objective function. In this study, MAR13082 was chosen as an objective function which is an artificial reaction created that generates biomass. As tumor cells need to grow and produce biomass to divide, this can be considered a suitable objective function for modeling metabolic fluxes. Once the optimal value of *f*_*max*_ was determined, parsimonious FBA was used to minimize internal metabolic fluxes. This provides a proxy for cells maximizing growth while minimizing nutrient consumption and use. Gurobi 9.0 was used to perform these optimizations.

#### Media constraint-based metabolic modeling

To simulate experimental cell culture conditions, the modeled external compartment contained metabolites detected in human blood. Exchange reactions (metabolite uptake reactions) for metabolites in this external compartment were opened only for those found in human blood, while all export reactions were opened. A radial diffusion model, as implemented in,^21^ was used to constrain metabolite uptake and model nutrient-limiting conditions. This model defines uptake constrains for each reaction according to the equation *U*_*i*_ = *αD*_*i*_*c*_*b*,*i*_ where *U*_*i*_ represents the uptake flux for metabolite *i*, Di represents the diffusion coefficient for metabolite *i* and *c*_*b*,*i*_ represents the concentration of metabolite *i* in the blood.

Blood metabolite concentrations were primarily derived from plasma metabolomics measurements from a large-scale cohort study of 8,413 healthy individuals using capillary electrophoresis-mass spectrometry.^64^ Diffusion coefficients were assigned in a metabolite-specific manner. Experimentally measured diffusion coefficients were used where available,^65^ including values reported for polar metabolites in biological fluids, while diffusion coefficients for the remaining small polar metabolites were estimated using a molecular mass-based linear model, as justified in.^66^ Diffusion coefficients for oxygen^67^ and albumin^68^ were obtained separately from the literature, with lipid diffusion approximated based on albumin because lipids are transported as larger lipid-associated complexes.

As comprehensive measurements of metabolite diffusivity and interstitial nutrient concentrations across different human tissues are currently unavailable, these parameters were not varied between tumor types. Instead, a common blood-derived nutrient environment was applied across all cancer types, allowing systematic comparison of tumor-specific metabolic adaptations under equivalent nutrient availability constraints. For a full justification of this model, please refer to.^21^

Values of *α* represent a function that is proportional to the inverse distance from the blood, with lower values representing those cells that are nutrient-starved and higher values representing cells with sufficient nutrients to support maximal growth. To determine optimal *α* values for simulating nutrient-replete and nutrient-deprived conditions, we empirically plotted various *α* values against the maximal achievable biomass across all models. Accordingly, we used *α* = 1 × 10^−5^ to simulate fluxes under nutrient deprivation and *α* = 2 × 10^−4^ to simulate fluxes under nutrient-replete conditions.

#### Transcription factor and pathway activity analysis

Transcription factor (TF) and pathway activities were inferred from TCGA transcriptomics data using DoRothEA, decoupleR, and PROGENy. DoRothEA was used to estimate TF activities based on regulon enrichment, while decoupleR provided a flexible framework for integrating multiple statistical methods to infer regulatory interactions. PROGENy was employed to assess pathway activity by leveraging curated footprints of pathway-responsive genes. All analyses were conducted in R, following the methodologies outlined in the respective package documentation.

#### Multi-omics machine learning for cancer survival prediction

Machine learning models were trained to predict survival outcomes using four separate datasets: clinical, fluxomics, transcriptomics, and genomics. Categorical variables were processed using one-hot encoding, while continuous variables were normalized using a combination of robust scaling and min-max (0–1) scaling. Each omics dataset was scaled independently to preserve intra-omics feature relationships (e.g., fluxomics and transcriptomics were normalized separately). All survival models were trained using right-censored overall survival data. Censored observations were retained throughout model training and evaluation by employing survival-specific learning algorithms and Uno’s concordance index, thereby allowing patients without an observed event to contribute appropriately to both model fitting and performance assessment.

To account for differences in survival distributions across cancer types, model training and testing splits were stratified by tumor type. Additionally, tumor type was incorporated as a covariate during model training to account for tissue-specific baseline survival differences and minimize prediction biases arising from uneven cancer type representation within TCGA. Multiple survival models were trained, including.

• Random Forest Survival
• Cox Elastic Net Regression
• Gradient Boosting Survival Analysis (GBSA)
• Kernel Survival Support Vector Machine (KSVM)

Each algorithm was evaluated across five independent train/test splits to assess the robustness of model performance. For each split, models were trained using 5-fold stratified cross-validation within the training set, maintaining stratification by tissue type and survival time to ensure balanced training cohorts. Final performance metrics were calculated across the independent test sets to account for variability introduced by sample partitioning. After model training, the best-performing models were analyzed for feature importance using their learned coefficients. The most informative features were selected to construct an ensemble survival model, integrating key predictors from multiple omics layers.

#### Transfections

Stable overexpression of EGFR and the corresponding negative control was achieved using the pCMV3-EGFR (HG10001-CY, Sino Biological) and pCMV3-untagged negative control vector (CV011, Sino Biological) plasmids that contain a hygromycin resistance gene. Cells were transfected using the FuGENE HD transfection reagent (Promega, Ref:E−2311) following the manufacturer’s recommendations. Briefly, 8 μL of FuGENE reagent was mixed with 2 μg of plasmid DNA in serum-free media to make a final volume of 100 μL and incubated for 10 min. The mixture was then added directly to Caco-2 cells seeded at a density of 500,000 cells/well in a 6-well plate. After 24 h, the medium was replaced with fresh medium to remove the transfection reagent. Following a further 24 h incubation, selection was carried out using 100 μg/mL hygromycin (Invitrogen, ref. 10687010) for two weeks. Stable overexpression was then assessed by western blotting.

#### Cell viability assays

To assess inhibition of *de novo* purine synthesis and purine salvage, the following inhibitors were used: mycophenolic acid (Sigma-Aldrich, M5255-50MG), mizoribine (MedChemExpress, HY-17470), forodesine (MedChemExpress, HY-16210), 6-thioguanine (MedChemExpress, HY-13765), azaserine (MedChemExpress, HY-B0919), pemetrexed (MedChemExpress, HY-10820), lometrexol (MedChemExpress, HY-14521).

Treatments with purine synthesis and salvage inhibitors were performed in the presence of 10% dialyzed FBS (GIBCO, 26400044) to avoid the presence of nucleosides and nucleobases. However, to maintain a physiological levels of purine salvage intermediates, 1 μM hypoxanthine (Sigma-Aldrich, H9377) and 100 nM adenine (Sigma-Aldrich, A8626) were supplemented in the medium. To determine cellular sensitivity to specific purine synthesis inhibitors, cells were seeded in 80 μL of medium at a density of 2,000 cells/well in a 96-well plate. The following day, cells were treated with inhibitors at 5x the final concentration in 20 μL of medium for experiments conducted in seeding medium. Thereafter, the medium was exchanged every 48 h to maintain the drug concentration. Following a 7-day incubation, cells were fixed with 60% TCA, and cell viability was assessed using the sulforhodamine B (Sigma-Aldrich, SRB; S1402) assay, as described previously.^69^

#### Liquid chromatography-mass spectrometry (LC-MS)-based metabolomics

Fo the nutrient-deprivation experiment, two million cells were seeded per 60-mm Petri dish and incubated for 48 h in nutrient-deprived medium in sextuplicate, allowing sufficient time for the cells to attach and resume proliferation. After this period, cells were placed in medium containing either 100% labeled D-Glucose-^13^C_6_ (Cambridge Isotope Laboratories, CLM-1396–1) or L-Glutamine-^13^C_5_ (Cambridge Isotope Laboratories, CLM-1822-H-0.1MG). Cells were then incubated for 8 h to allow sufficient flux of the labeled substrates into the TCA cycle. Cells were washed once with ice-cold PBS, snap-frozen in liquid nitrogen and placed on ice. Metabolites were extracted with 300 μL of extraction buffer (methanol:acetonitrile:water, 40:40:20, pre-chilled at −20°C). Samples were then centrifuged for 10 min at +4°C and 10,000 RPM and the supernatant was transferred to screw-cap tubes for long-term storage at −80°C. Subsequently, 100 μL of the metabolite solution was mixed with 100 μL of acetonitrile, vortexed briefly, centrifuged for 10 min at +4°C, 10,000 RPM and finally transferred to LC-MS V-shaped vials for analysis.

For asparagine synthesis labeling, one million cells were seeded per 60-mm Petri dish and incubated for 24 h in full medium in triplicate. After this period, cells were placed in medium containing 100% amide-^15^N L-Glutamine (Cambridge Isotope Laboratories, NLM-557–1). Cells were then incubated for 8 h to allow sufficient labeling of glutamine into asparagine. Cells were washed once with ice-cold PBS, snap-frozen in liquid nitrogen and placed on ice. Metabolites were extracted with 300 μL of extraction buffer (methanol:acetonitrile:water, 40:40:20, pre-chilled at −20°C). Samples were then centrifuged for 10 min at +4°C, 10,000 RPM, and the supernatant was transferred to screw-cap tubes for long-term storage at −80°C. Subsequently, 100 μL of the metabolite solution was mixed with 100 μL of acetonitrile, vortexed briefly, centrifuged for 10 min at +4°C and 10,000 RPM, and finally transferred to LC-MS V-shaped vials for analysis.

For GSH synthesis labeling, 600,000 cells were seeded per 60-mm Petri dish and incubated for 24 h in full medium in quadruplicate. Following this period, cells were cultured under the indicated experimental conditions with the appropriate siRNA-mediated knockdown and bile acid supplementation. Subsequently, cells were placed in medium containing 100% L-Cystine (3,3′-^13^C_2_, 99%) (Cambridge Isotope Laboratories, CLM-520–0.25). Cells were then incubated for 4 h to allow sufficient labeling of cystine into GSH. Medium was collected and immediately placed in extraction buffer (methanol:acetonitrile:water, 40:40:20, pre-chilled at −20°C) supplemented with 10 mM N-ethylmaleimide (NEM) to preserve the intracellular glutathione redox state. After the medium was extracted, cells were washed once with ice-cold PBS, supplemented with 30 mM NEM, snap-frozen in liquid nitrogen, and placed on ice. Metabolites were extracted with 300 μL of extraction buffer. Samples were then centrifuged for 10 min at +4°C, 10,000 RPM, and the supernatant was transferred to screw-cap tubes for long-term storage at −80°C. Subsequently, 100 μL of the metabolite solution was mixed with 100 μL of acetonitrile, vortexed briefly, centrifuged for 10 min at +4°C and 10,000 RPM, and finally transferred to LC-MS V-shaped vials for analysis.

Metabolomics analysis was performed using a UHPLC-Q/TOF system. Hydrophilic interaction liquid chromatography was carried out on an InfinityLab Poroshell 120 HILIC-z column (2.7 μm, 2.1 mm × 100 mm, PEEK-lined; Agilent: 675775–924) maintained at 50°C. Samples were maintained at 4°C in an autosampler, and the injection volume was set to 3 μL. Metabolites were eluted through the column at a flow rate of 0.250 mL/min using solvent A, comprising 10 mM ammonium acetate in water (pH 9) supplemented with 2.5 μM InfinityLab Deactivator Additive and solvent B, comprising 10 mM ammonium acetate in acetonitrile:water (85:15, v/v; pH 9) supplemented with 2.5 μM InfinityLab Deactivator Additive. The following gradient was used for elution: 0 min, 96%B; 2 min, 96%B; 5.5 min, 88%B; 8.5 min, 88%B; 9 min, 86%B; 14 min, 86%B; 17 min, 82%B; 23 min, 65%B; 24 min, 65%B; 24.5 min, 96%B; 26 min, 96%B, followed by a 3-min post-run re-equilibration period before injection of the next sample. MS analysis was performed on an Agilent 6546 LC/Q-TOF mass spectrometer in negative polarity mode. Dynamic mass axis correction was achieved by infusion of a reference mass solution using an isocratic pump set at a flow of 1 mL/min. MS settings were as follows: ionization mode: AJS; gas temperature, 225°C; drying gas, 13 L/min; nebuliser pressure, 35 psi; sheath gas temperature, 350°C; sheath gas flow, 12 L/min; capillary voltage, 3,500 V; fragmentor voltage, 125 V; skimmer voltage, 45 V; and octopole 1 RF voltage, 750 V. Data were acquired at a rate of one spectrum/sec over an m/z range of 60–1,600. Agilent MassHunter Workstation Data Acquisition software was used for data acquisition. For data analysis, Agilent Profinder B.8.0.00 was used with a library of pre-determined retention times ( ±0.20 min) and accurate mass ( ±15 ppm) for metabolites of interest.

Standards of asparagine (Cat# A4159, Sigma-Aldrich), aspartate (Cat# A8949, Sigma-Aldrich), fumarate (Cat# 47910, Sigma-Aldrich), and malate (Cat# 02288, Sigma-Aldrich) were prepared as 20 mM stock solutions in LC-MS grade water. Working solutions at concentrations of 200 μM and 2 μM were subsequently prepared by diluting the stocks in a solvent mixture of acetonitrile, methanol, and water (40:40:20, v/v/v).

#### Seahorse analysis

Cells’ metabolic behavior was assessed using the Seahorse XFe96 Analyzer (Agilent), which measures oxygen consumption rate (OCR) and extracellular acidification rate (ECAR) under basal or treated conditions. Three days before the experiment, OE33, AGS, CAL27 or HCT116 (2,000 cells/well) were seeded in an XFe96 cell culture plate (Agilent, ref. 101085–004) and kept overnight at 37°C in a 5% CO_2_ incubator. The following day, media was exchanged to allow cells to be treated with bile acids.

The FA oxidation (FAO) assay (Agilent, ref. 102720–100) was employed to evaluate the ability of cells to utilize palmitate – a representative long-chain FA and a key product of *de novo* lipogenesis – as a substrate for mitochondrial respiration. One day before the Seahorse FAO assay, culture medium was replaced with substrate-limited medium consisting of DMEM lacking glucose, glutamine, and pyruvate (Agilent; Part no. 103575–100), supplemented with 1 mM glucose, 1 mM glutamine, 1% FBS, and 0.5 mM carnitine. Cells were cultured overnight under these conditions to reduce intracellular nutrient stores and promote dependency on FAO before measurement of OCR. On the day of the assay, the medium was replaced with Krebs–Henseleit buffer (KHB) comprising 110 mM NaCl, 4.7 mM KCl, 2 mM MgSO_4_, 1.2 mM NaH_2_PO_4_, 0.5 mM L-carnitine, 5 mM HEPES, and 2.5 mM glucose in ultrapure water (pH 7.4 ± 0.1). Cells were washed twice with KHB and incubated for 1 h at 37°C in a non-CO_2_ incubator. 15 min before the assay, selected wells were supplemented with 40 μM etomoxir. Immediately before the assay, 20 μL per well of palmitate–BSA conjugate was loaded into the sensor cartridge ports for substrate delivery. Following baseline measurements, cells were sequentially treated with oligomycin (1.5 μM; Sigma-Aldrich, 75351), carbonyl cyanide 4-(trifluoromethoxy)phenylhydrazone (FCCP; 1 μM; Sigma-Aldrich, C2920), and rotenone (Sigma-Aldrich, R8875) and antimycin A (Sigma-Aldrich, A8674) (0.5 μM each) to determine ATP-linked respiration, maximal respiratory capacity, and non-mitochondrial respiration, respectively. Results were normalized using the SRB protocol. Cells were fixed with 60% TCA, and cell viability was assessed using the sulforhodamine B (Sigma-Aldrich, SRB; S1402) assay, as described previously.^70^

#### Measurement of intracellular ROS

Intracellular reactive oxygen species (ROS) levels were quantified by flow cytometry using the CM-H_2_DCFDA probe (ThermoFisher Scientific; C6827), in accordance with the manufacturer’s protocol. Cells were seeded at 1 × 10^6^ cells per well in 60-mm plates and treated the following day for 48 h with the appropriate bile acid concentration indicated in the Figure S5 legend. Following treatment, cells were incubated with 5 μM CM-H_2_DCFDA in phenol red–free, serum-free DMEM for 30 min at 37°C in the dark. Cells were then washed, trypsinized, and resuspended in 500 μL Hank’s Balanced Salt Solution (HBSS) containing NucBlue Fixed Cell ReadyProbe reagent for nuclear staining. Samples were analyzed within 30 min on a BD LSRII flow cytometer. Debris and cell aggregates were excluded using forward- and side-scatter gating, and dead cells were excluded by detecting NucBlue fluorescence (excitation at 404 nm; 450/50 nm emission filter). ROS-associated FITC fluorescence was recorded using excitation at 488 nm and a 530/30 nm band-pass filter.^69^

### Quantification and statistical analysis

#### Computational analyses

For inferring relative up- or down-regulation, one-sided Wilcoxon signed-rank tests were used, as described in.^43^ For determining the significance of CNV changes between groups that upregulated metabolic functions, such as asparagine synthesis, Fischer’s exact-test was used. For comparisons of flux pathway activity comparison between groups of interest, a one-way ANOVA followed by Tukey’s post hoc test was used. Spearman rank correlation coefficients and associated *p* values were calculated in R using the cor.test function. Survival analyses were performed using the Cox proportional hazards model, and differences between groups were assessed using the log rank test. Analyses were conducted in R (version 4.5.1) using the *survival* package. Relevant details for statistical tests are provided in the figure legends.

#### Experimental validations

One- or two-way ANOVA was used to evaluate statistical significance, as indicated in the respective figure legends, followed by Tukey’s post hoc test. For experimental results, the ‘n’ for each experiment is provided in the figure legends and represents independently generated samples for *in vitro* experiments or wells for cell-based assays. Bar graphs present the mean ± standard error of the mean (s.e.m.). No statistical methods were used to determine sample size, and missing data were excluded from the analysis performed in this manuscript. Statistical tests were performed using GraphPad Prism (version 10.6.1).

## Supplemental information

Document S1. Figures S1–S15

Table S1. Normalized metabolite abundance values from stable isotope tracing experiments

Table S2. Cell line information, culture conditions, and experimental details

Table S3. TCGA cancer type abbreviations and corresponding tumor classifications

Document S2. Article plus supplemental information
