# Supplementary material for: FluxAtlas is a pan-cancer metabolic flux atlas that identifies tumor-selective dependencies and prognostic biomarkers
Source: Cell Rep. 2026 Sep 24;45(10):118029. doi: 10.1016/j.celrep.2026.118029 (PMC13714176; doi:10.1016/j.celrep.2026.118029)
Supplement: Supplementary file 1 [file mmc1.pdf]

Cell Reports, Volume *45*

Supplemental information

FluxAtlas is a pan-cancer metabolic flux atlas that identifies tumor-selective dependencies and prognostic biomarkers

Manas Kohli, Aurélien Tripp, Anastasiia Shcherbakova, and George Poulogiannis

Figure S1.

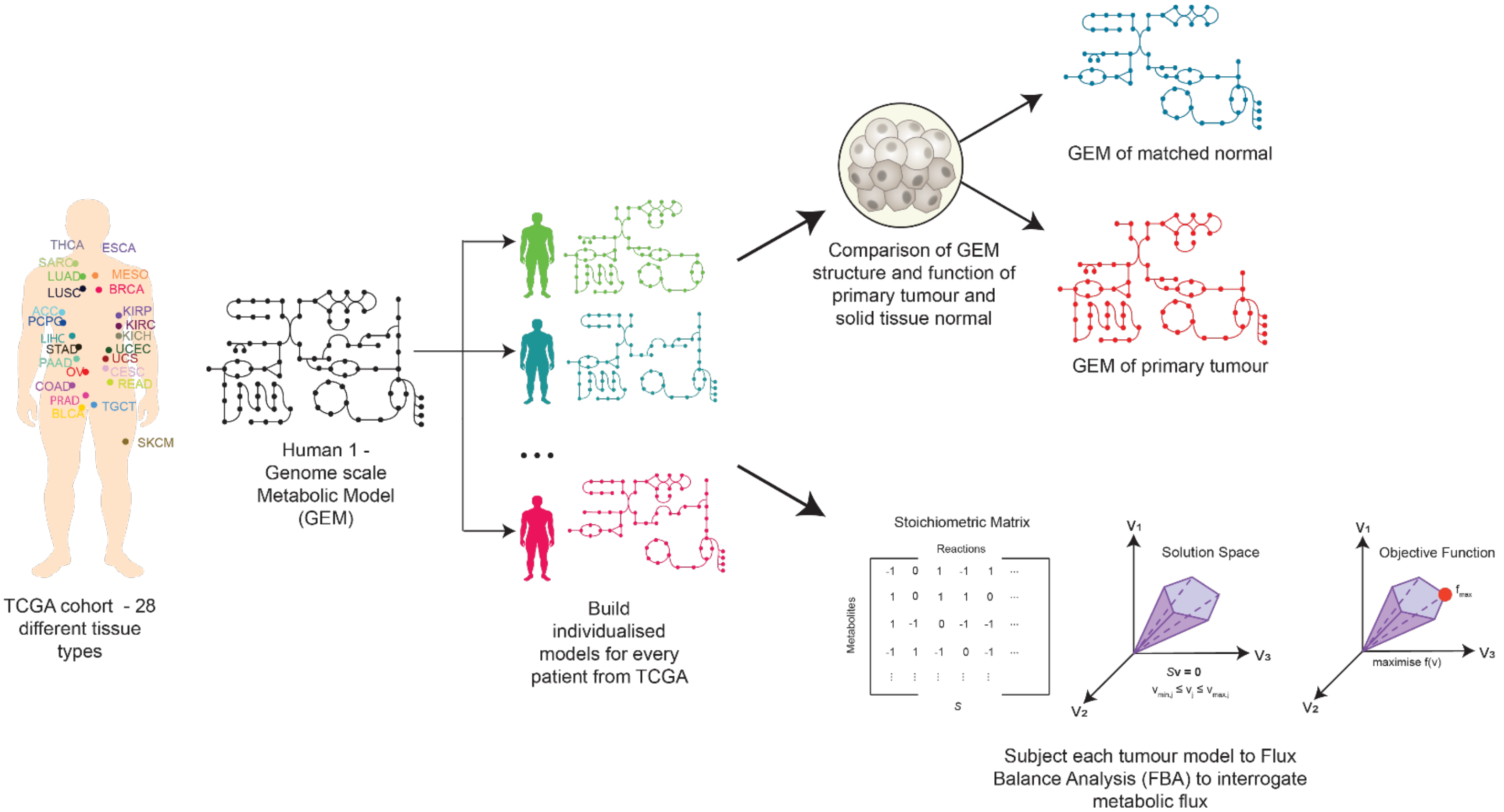

Figure S1. Construction of a pan-cancer metabolic flux atlas from patient-specific genome-scale metabolic models. This study includes 9,554 patients across 28 tissue types. Transcriptomic data obtained from patients' tumors and matched normal tissues were used to generate genome-scale metabolic models (GEMs).

Figure S2.

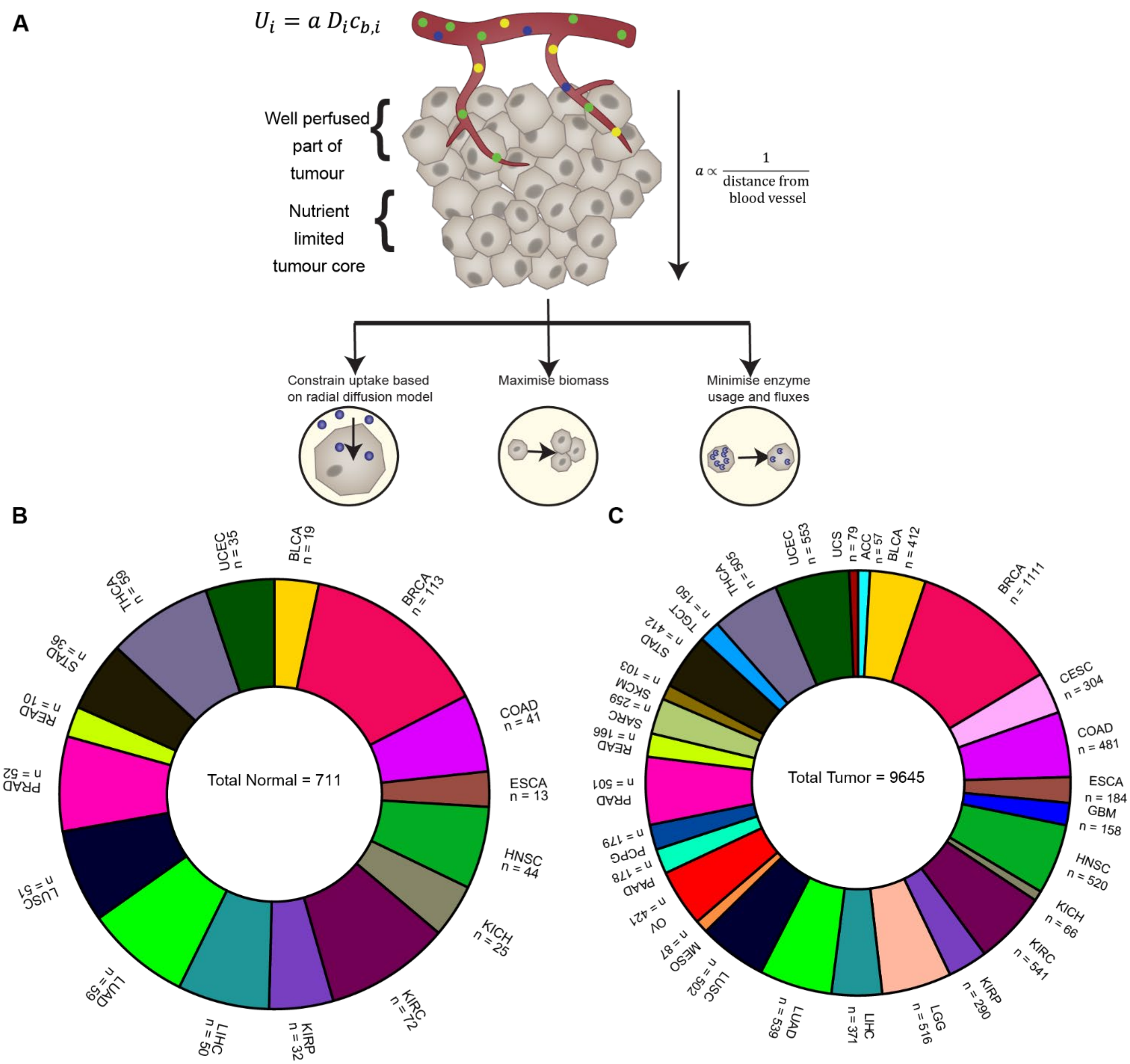

Figure S2. Overview of the modelling approach for flux balance analysis carried out in this study.

(**A**) Schematic showing relevant model constraints applied in FBA. Each nutrient uptake was constrained according to the equation $U_i = \alpha D_i c_{b,i}$, where D_i represents the diffusion constant for each metabolite, $c_{b,i}$ represents its concentration in the blood, and α represents a constant that is an inverse function of the distance from the nutrient source, which in this case is the blood. Using appropriate values of α allows simulation of fluxes under nutrient-deprived and nutrient-replete conditions. In addition, models were enzyme-constrained, and parsimonious FBA was applied to

generate more physiologically relevant fluxes by minimizing internal fluxes. (**B**) Chart showing the tissue distribution and number of samples for normal samples analyzed. (**C**) Chart showing the tissue distribution and number of tumor samples analyzed.

Figure S3. Related to Figure 1.

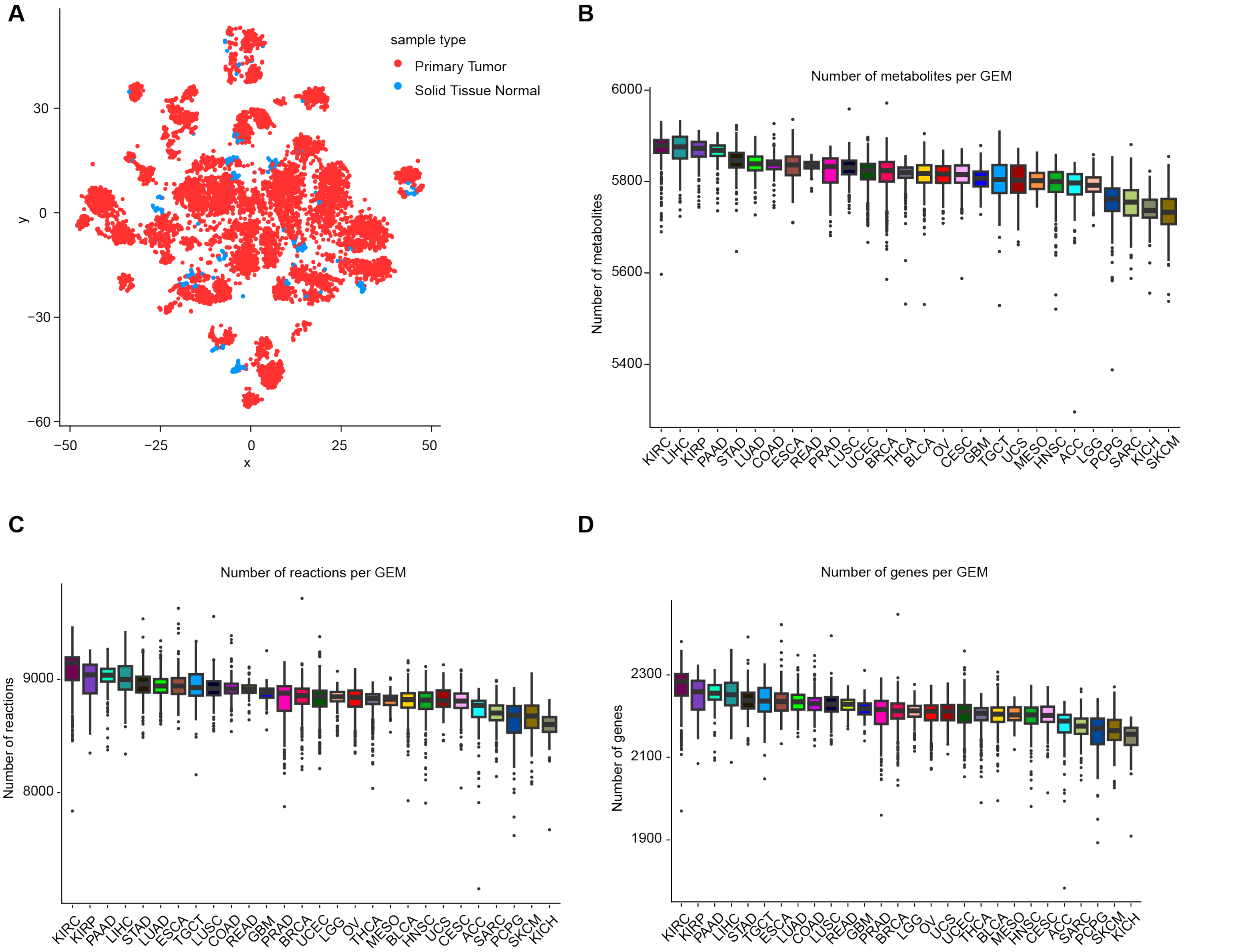

Figure S3: Properties of metabolic networks generated by tINIT.

(**A**) t-SNE plot highlighting the distribution of normal samples and tumor samples, showing the overall dispersion of normal samples. (**B**)–(**D**) Boxplots depicting numbers of (B) metabolites, (C) reactions and (D) genes present in tumor GEMs, stratified by tumor type.

Figure S4. Related to Figure 1.

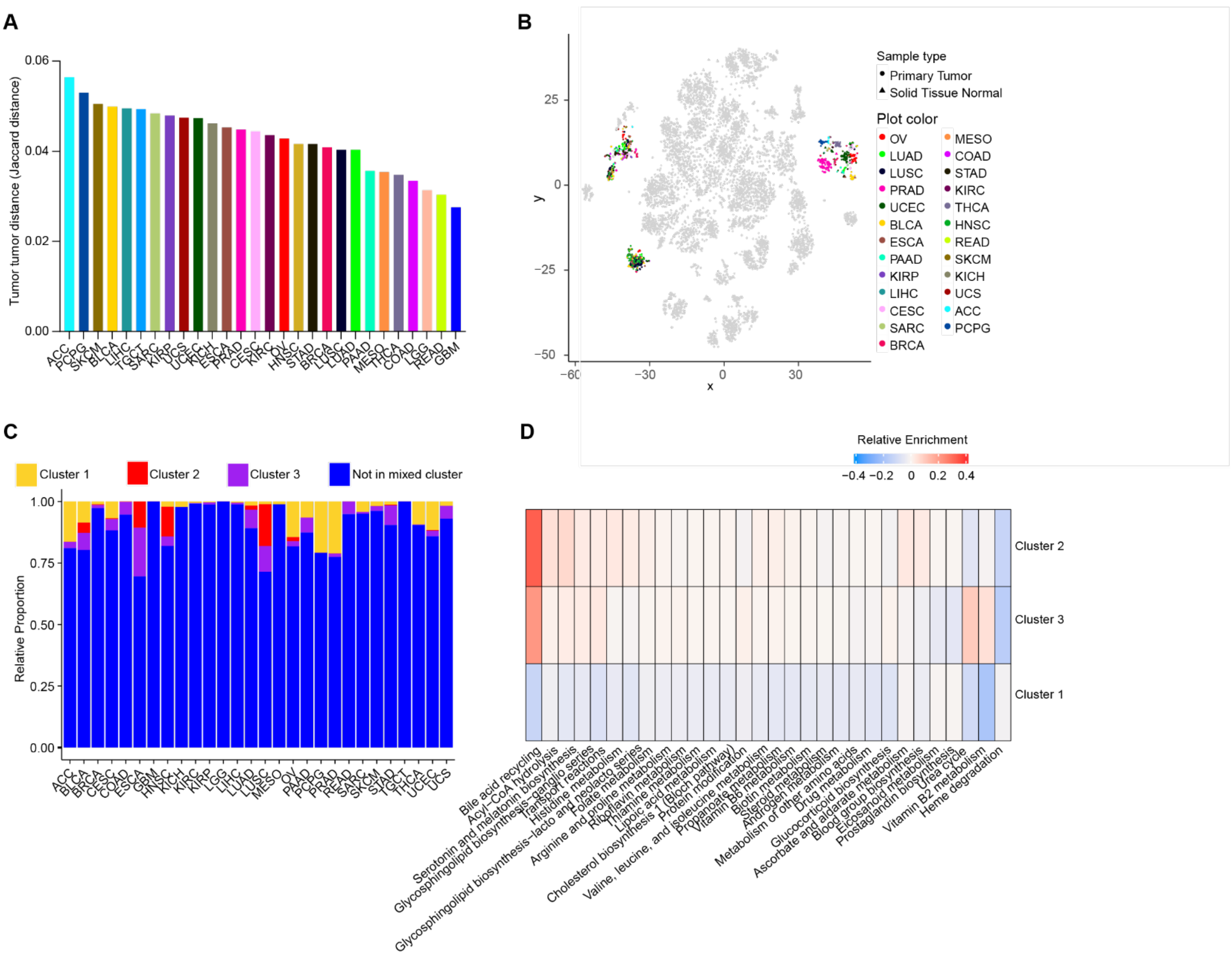

Figure S4: Clustering of samples based on their metabolic activity profiles generated using Flux Balance Analysis highlights both tissue-dependent and tissue-independent clusters.

(**A**) Average Jaccard distance between each tumor GEM and all other tumor GEMs within the same tissue type. (**B**) t-SNE plot highlighting clusters of samples composed of multiple tissue types. (**C**) Stacked bar chart showing that these mixed clusters comprise different tissue types. (**D**) Heatmap showing metabolic pathways enriched in each cluster compared with the remaining samples.

Figure S5. Related to Figure 1.

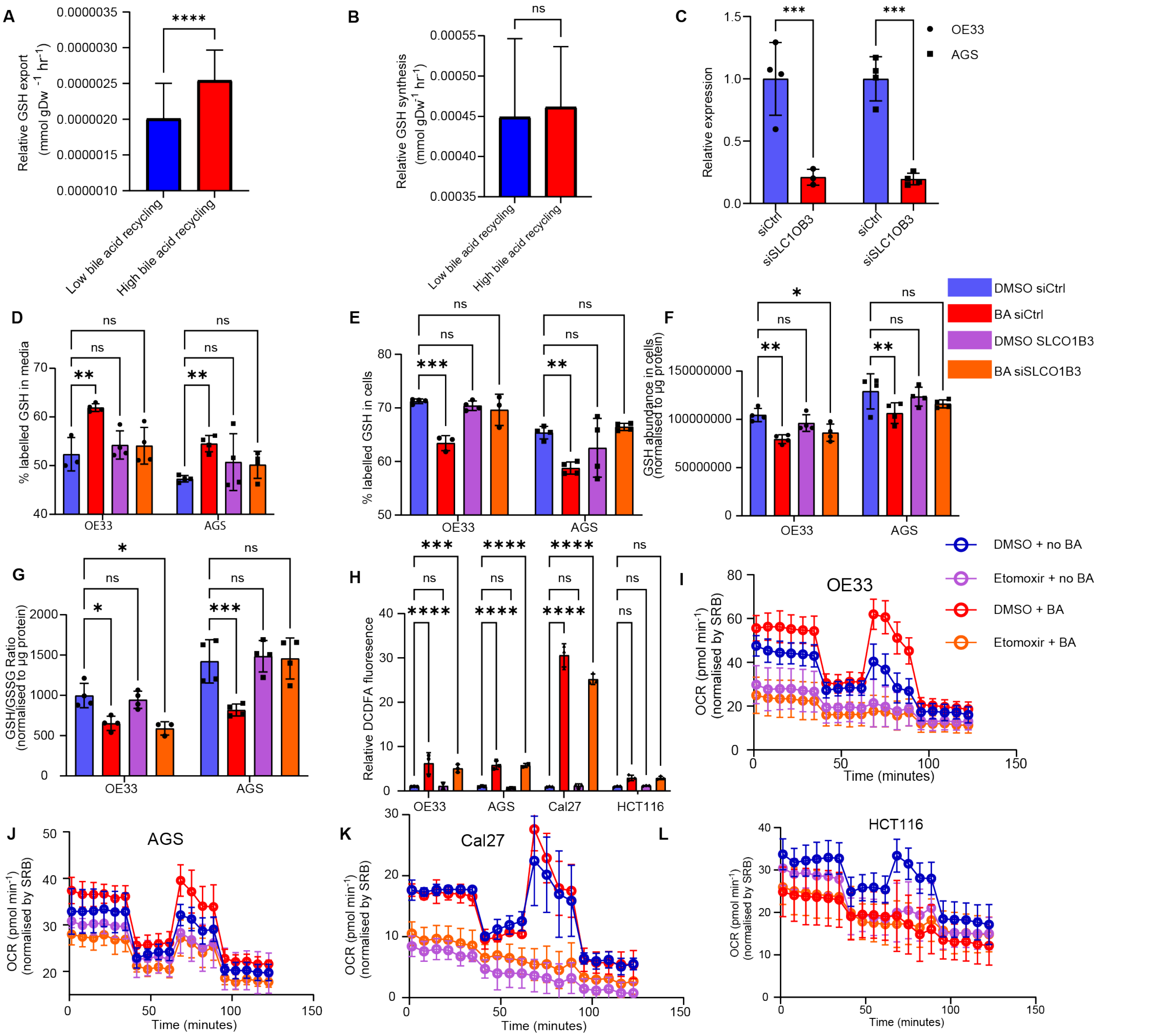

Figure S5: Bile acid transport promotes altered glutathione localization, redox imbalance, and tissue-specific fatty acid oxidation

(**A**) Comparison of predicted glutathione (GSH) export flux between tumors with enriched bile acid (BA) recycling and tumors with low BA recycling. Total GSH export flux was calculated by summing the fluxes through all reactions contributing to extracellular GSH transport for each patient-specific FBA model. (**B**) Comparison of predicted GSH biosynthesis flux between tumors with enriched BA recycling and tumors with low BA recycling. Total GSH synthesis flux was calculated by summing the fluxes through reactions contributing to cytoplasmic GSH production. (**C**) Quantitative PCR analysis of *SLCO1B3* mRNA expression following siRNA-mediated knockdown compared with non-targeting control siRNA.

Relative expression was determined using the $2^{-\Delta\Delta Ct}$ method after normalization to housekeeping gene expression. (**D**) Extracellular labelled GSH abundance measured in cell culture medium following L-Cystine (3,3′-$^{13}C_2$) stable isotope tracing in OE33 and AGS cells treated with control siRNA (siCtrl) or *SLCO1B3* siRNA (siSLCO1B3), in the presence or absence of BA supplementation. (**E**) Intracellular labelled GSH abundance measured from cell extracts following L-Cystine (3,3′-$^{13}C_2$) tracing under control and *SLCO1B3* knockdown conditions, with or without BA supplementation. (**F**) Total intracellular GSH abundance measured by LC-MS and normalized to cellular protein content across the indicated experimental conditions. (**G**) Intracellular GSH/GSSG ratio measured by LC-MS and normalized to cellular protein content to assess changes in cellular redox state following BA supplementation and *SLCO1B3* knockdown. (**H**) Quantification of intracellular reactive oxygen species (ROS) levels determined by CM-H_2DCFDA staining and flow cytometry. Values represent the geometric mean fluorescence intensity of CM-H_2DCFDA across experimental conditions. (**I**-**L**) Seahorse fatty acid oxidation (FAO) stress test measuring oxygen consumption rate (OCR) following palmitate supplementation in OE33 (**I**), AGS (J), CAL27 (K), and HCT116 (L) cells to determine the effect of BA exposure on FAO across different tumor contexts. For all experiments, cells were treated with either control siRNA (siCtrl) or *SLCO1B3*-targeting siRNA (siSLCO1B3) and supplemented with DMSO vehicle control or 50 μM chenodeoxycholic acid (CDCA), where indicated. Data in (C-G) represent mean values ± s.e.m. from n = 4 biological replicates; data in (H) represent mean values ± s.e.m. from n = 3 biological replicates; and data in (G–J) represent mean values ± s.e.m. from n = 5 or 6 biological replicates. ns, not significant; **P* < 0.05; ***P* < 0.01; ****P* < 0.001; ****P < 0.0001. *P* values in (A-B) were calculated using Student's t-test, while those in (C–H) were calculated using two-way ANOVA with Tukey's post-hoc test.

Figure S6. Related to Figure 1

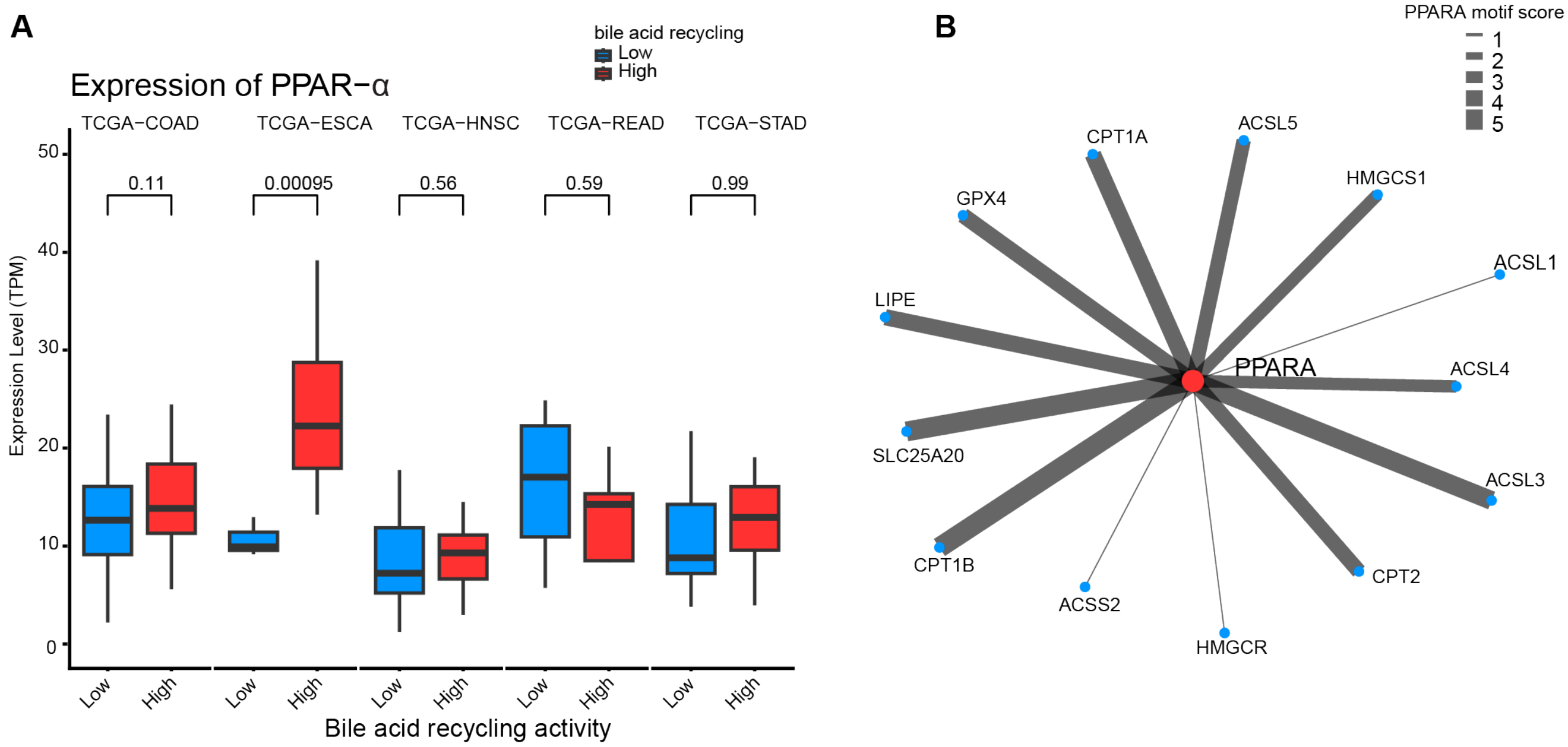

Figure S6: Bile acid recycling is associated with altered redox homeostasis and PPARα-mediated lipid metabolic rewiring

(**A**) Comparison of *PPARA* expression between tumors with enriched BA recycling and tumors with low BA recycling across gastrointestinal tumor types. (**B**) STRING network analysis of lipid metabolism-associated genes identified from differential FBA analysis. Genes were annotated based on predicted PPARα transcriptional regulation using promoter motif enrichment analysis, with edge weight indicating the strength of evidence supporting PPARα-associated regulation. *P* values in (A) were calculated using Student's t-test, and the exact *P* values are reported in (A).

Figure S7. Related to Figure 2.

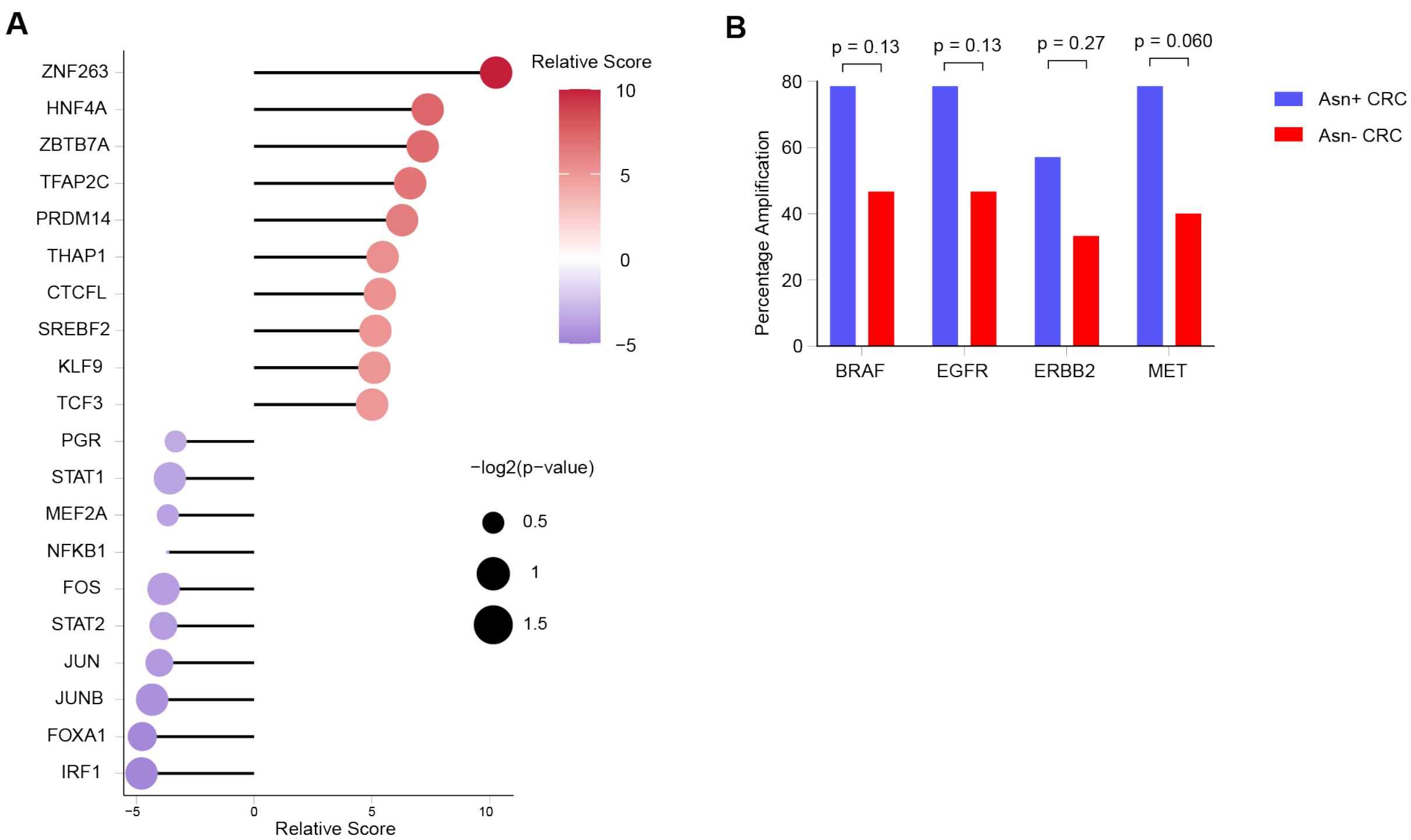

Figure S7: Associations between RTK-RAS signalling and asparagine synthesis.

(**A**) Bubbleplot of transcription factor activity comparing colorectal tumors that upregulate asparagine synthesis with those that do not in TCGA. (**B**) Bar plot of copy-number gain/amplification in key genes in the RTK-RAS pathway, comparing colorectal tumors that upregulate asparagine synthesis with those that do not in TCGA. *P* values in (A) were computed using two-sided *t*-tests on TF activity coefficients inferred by decoupleR (ULM), and those in (B) were computed using Fisher's exact test.

Figure S8. Related to Figure 3.

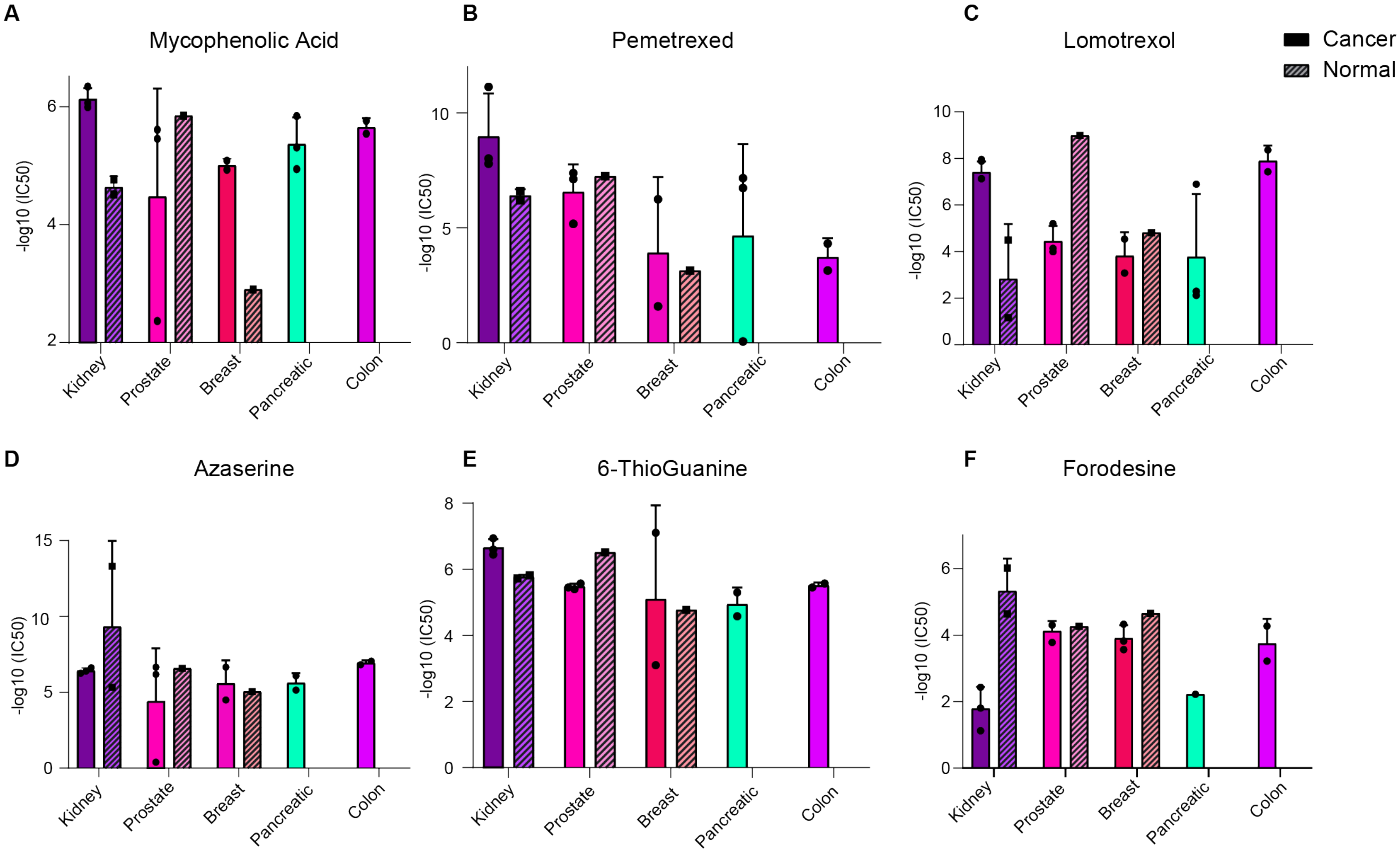

Figure S8: Sensitivity profiles of cancer and untransformed cell lines to different metabolic inhibitors.

(**A-F**) Bar plots of IC50 values calculated from dose-response experiments for various cell lines. Sensitivity scores are reported as -log10(IC50). For each cell line, a single IC50 value was calculated from a dose-response experiment conducted in biological triplicate (n=3). Data points correspond to the reported IC50 values for different cell lines. Purine synthesis inhibitors included (**A**) mycophenolic acid (MPA), (**B**) pemetrexed, (**C**) lomotrexol, (**D**) azaserine, (**E**) 6-thioguanine, while (**F**) forodesine was used as a purine salvage inhibitor.

Figure S9. Related to Figure 4.

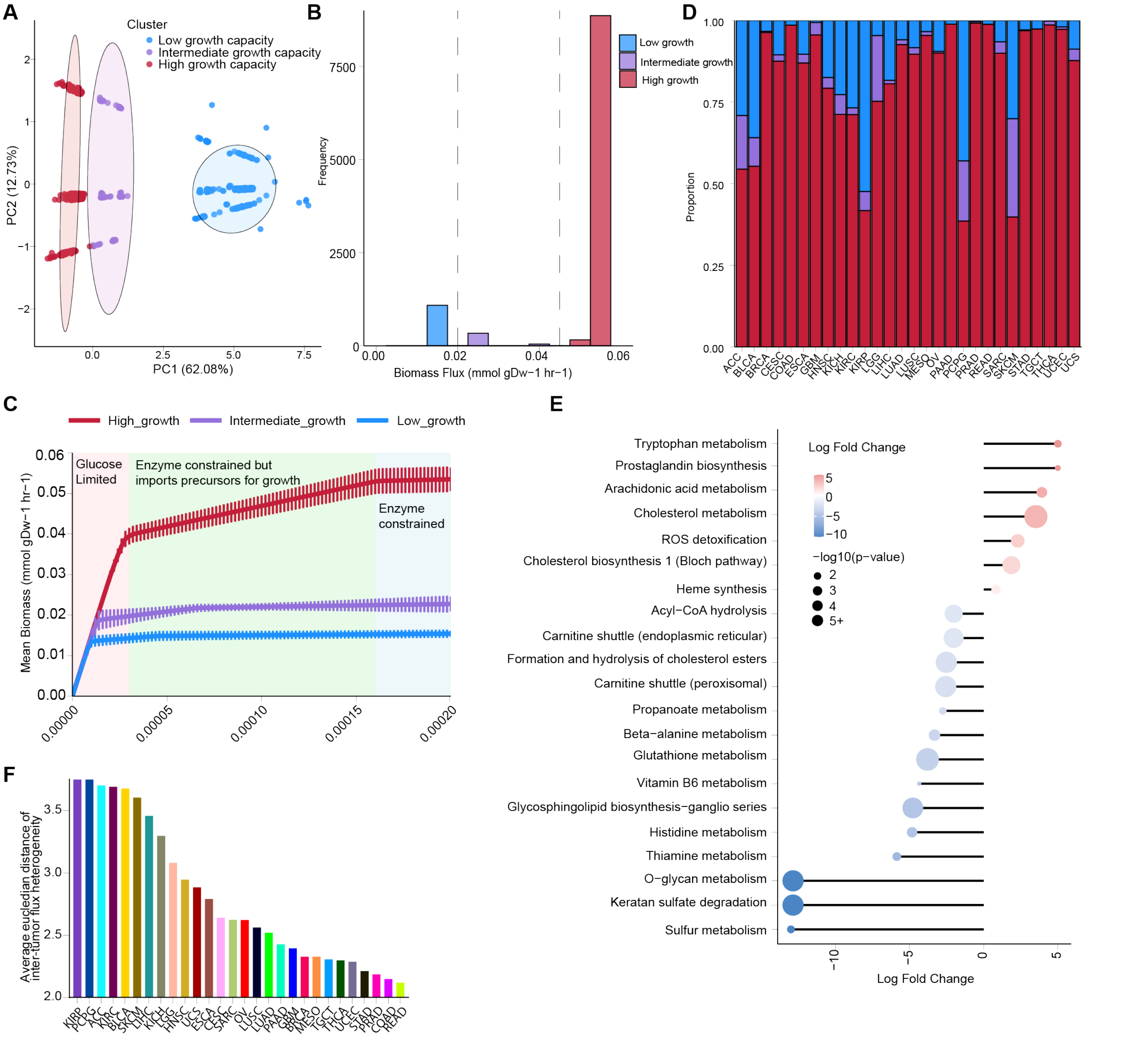

Figure S9: Unsupervised analysis of metabolic fluxes across TCGA.

(**A**) PCA plot of metabolic flux demonstrating the presence of three clusters. (**B**) Histogram of biomass flux delineating the different clusters. (**C**) Stacked bar chart showing that samples in the low and intermediate intracellular growth groups predominantly belong to specific tissue types. (**D**) Line chart comparing biomass flux across different values of α that serves as a proxy for nutrient availability. Samples from the intermediate and high growth groups cannot grow to the same extent, even under high nutrient availability. (**E**) Lollipop chart comparing metabolic fluxes in tumors from the low intracellular growth group with those from the high intracellular growth group. (**F**) Average Euclidean

distance plot comparing the fluxes of each sample with those of all other samples within specific tissue types. *P* values in (E) were calculated using two-way ANOVA with Tukey's post-hoc test.

Figure S10. Related to Figure 4.

A

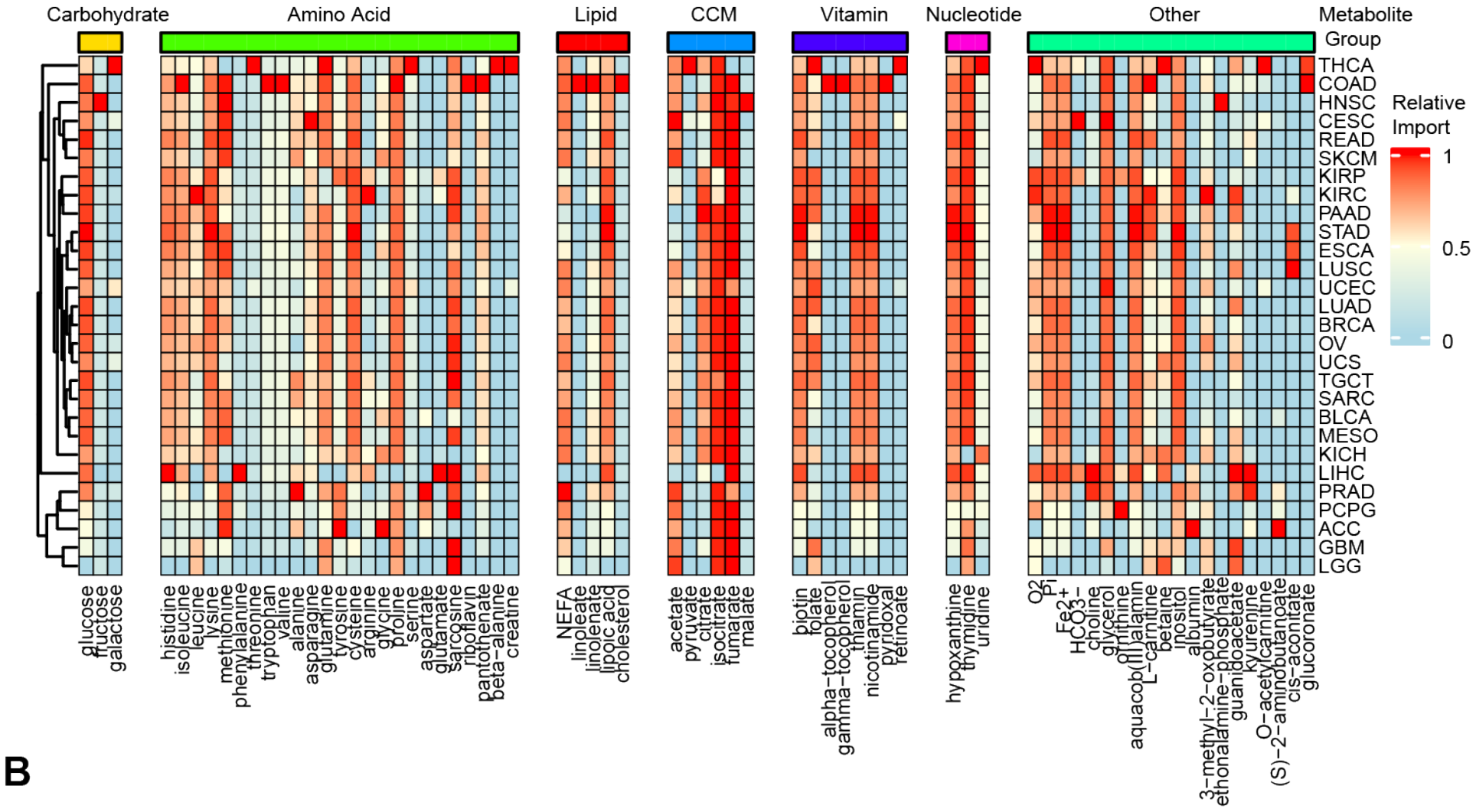

B

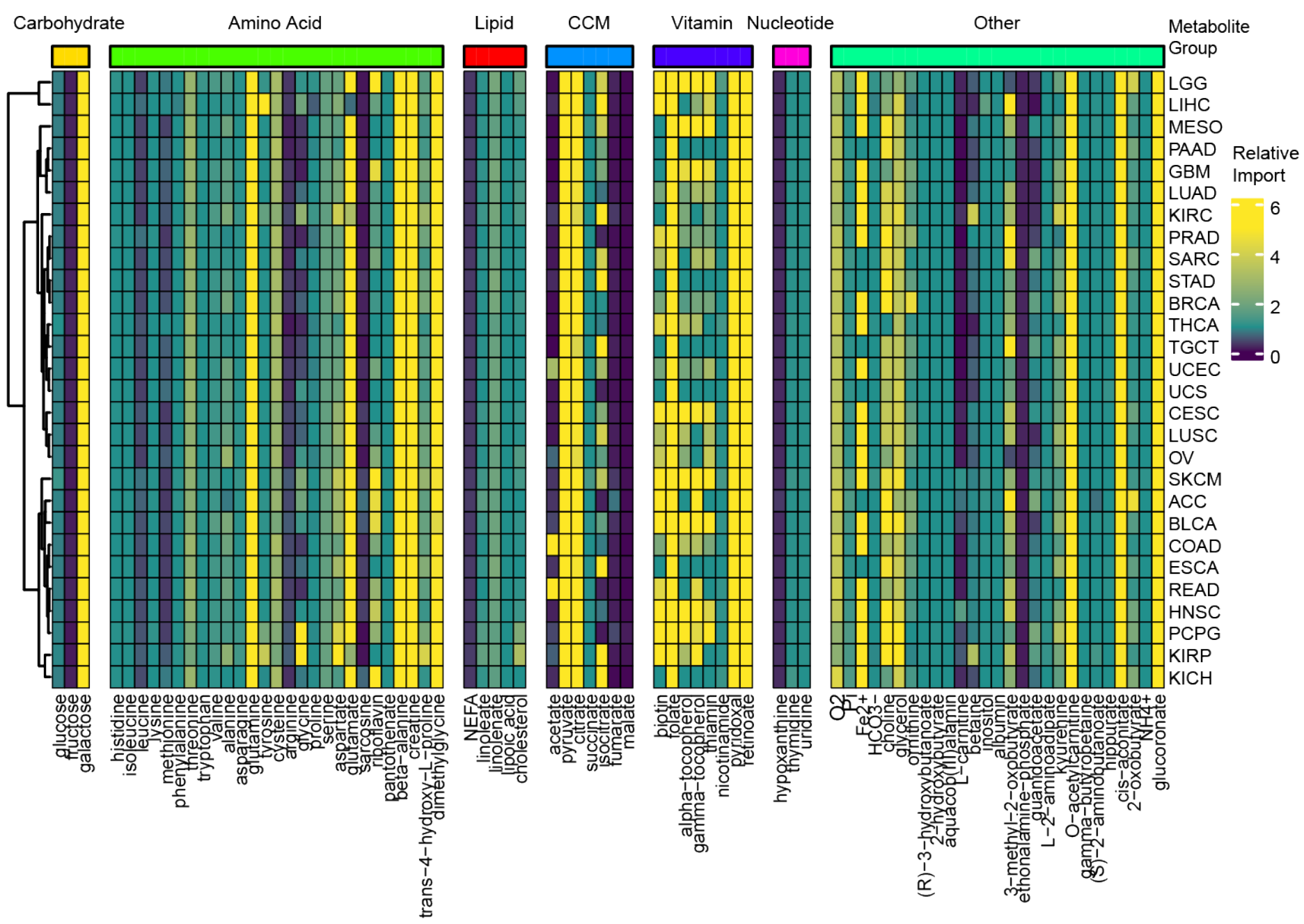

Figure S10: Relative nutrient import fluxes under nutrient-replete and nutrient-deprived conditions.

(**A**) Heatmap depicting relative flux through different import reactions across tissue types. Individual fluxes through each import reaction were averaged for each tissue type. For each metabolite, these values were scaled from 0 to 1. Metabolites were subsequently grouped according to their broad categories. (**B**) Heatmap showing metabolites whose import increased or decreased under nutrient-deprived conditions compared with nutrient-replete conditions. Import fluxes under both nutrient-deprived and nutrient-replete conditions were normalized to relative biomass.

Figure S11. Related to Figure 4.

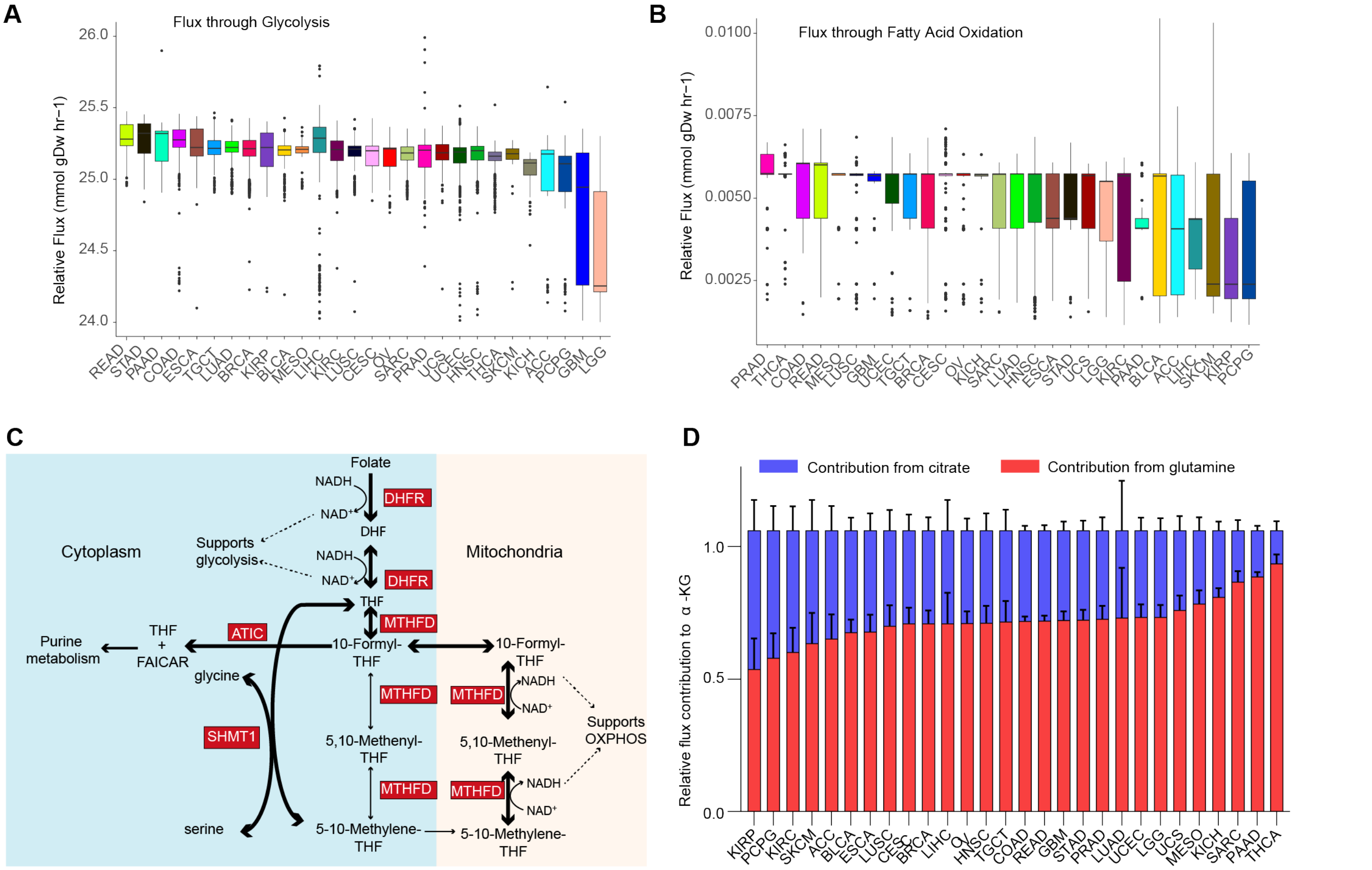

Figure S11: Adaptation to nutrient deprivation involves coordinated changes that are shared across tumor types but are not always universal.

(**A**) Boxplots depicting glycolytic flux across all tissue types, ordered from highest to lowest mean. (**B**) Boxplots depicting fatty acid oxidation flux across all tissue types, ordered from highest to lowest mean. (**C**) Schematic depicting changes in folate metabolism and serine-glycine metabolism under nutrient deprivation, allowing the continued synthesis of purines, oxidizing equivalents in the cytoplasm, and reductants in the mitochondria to support cellular metabolism. Reactions with increased flux under nutrient-deprived conditions are represented by thicker arrows, whereas reactions with thinner arrows have reduced flux under nutrient deprivation. (**D**) Barplot showing the relative contribution of citrate or glutamine to mitochondrial α-ketoglutarate levels.

Figure S12. Related to Figure 6.

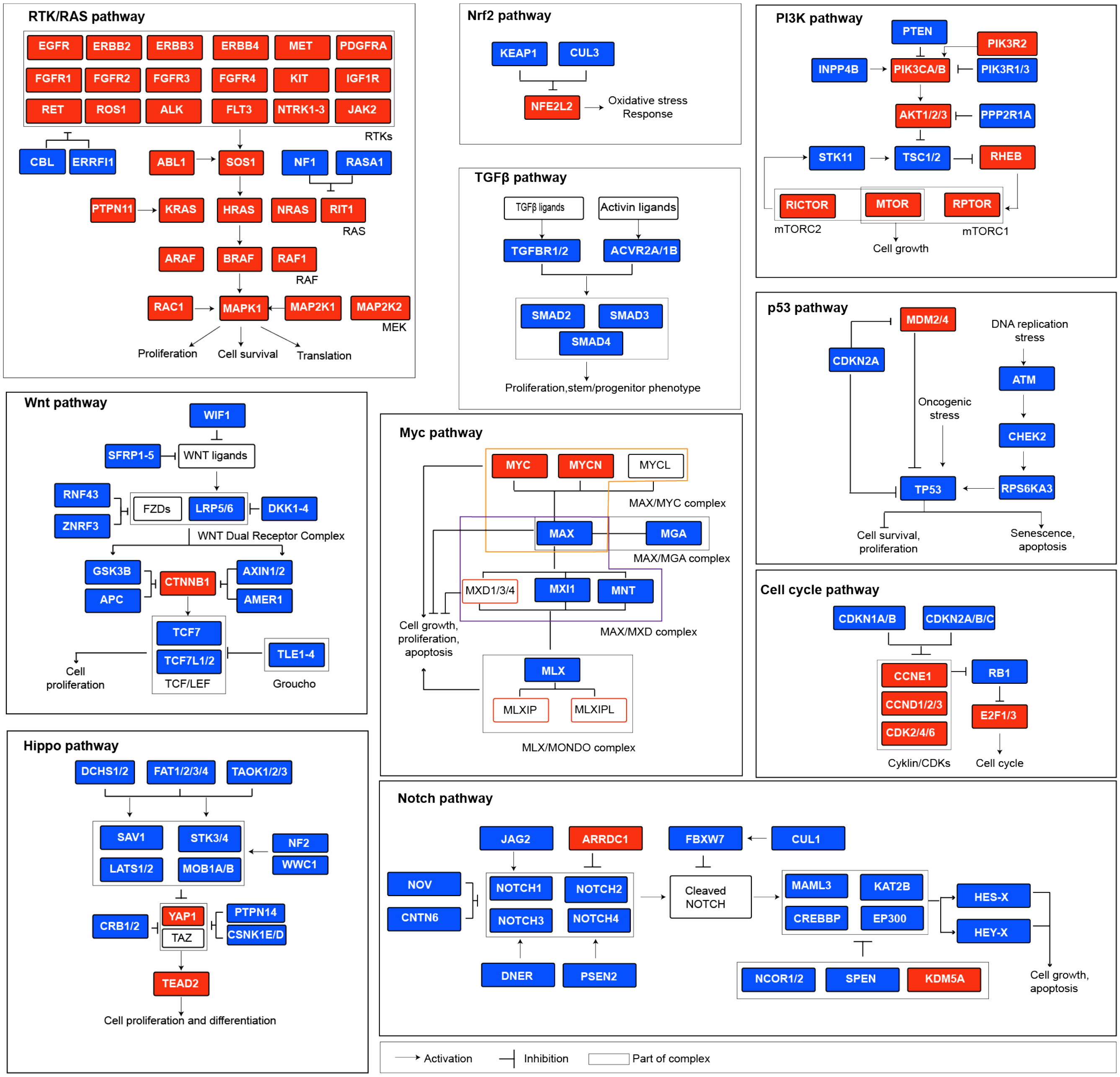

Figure S12: Major pathways driving tumor formation and growth, adapted from Sanchez-Vega et al. Oncogenes are shown in red and tumor suppressor genes are shown in blue. Alterations were defined as the presence of driver mutations, significant copy-number changes, changes in DNA methylation, or fusions that alter driver-gene activity. A tumor was considered altered if it possessed an alteration in at least one gene within a given pathway.

Figure S13. Related to Figure 6.

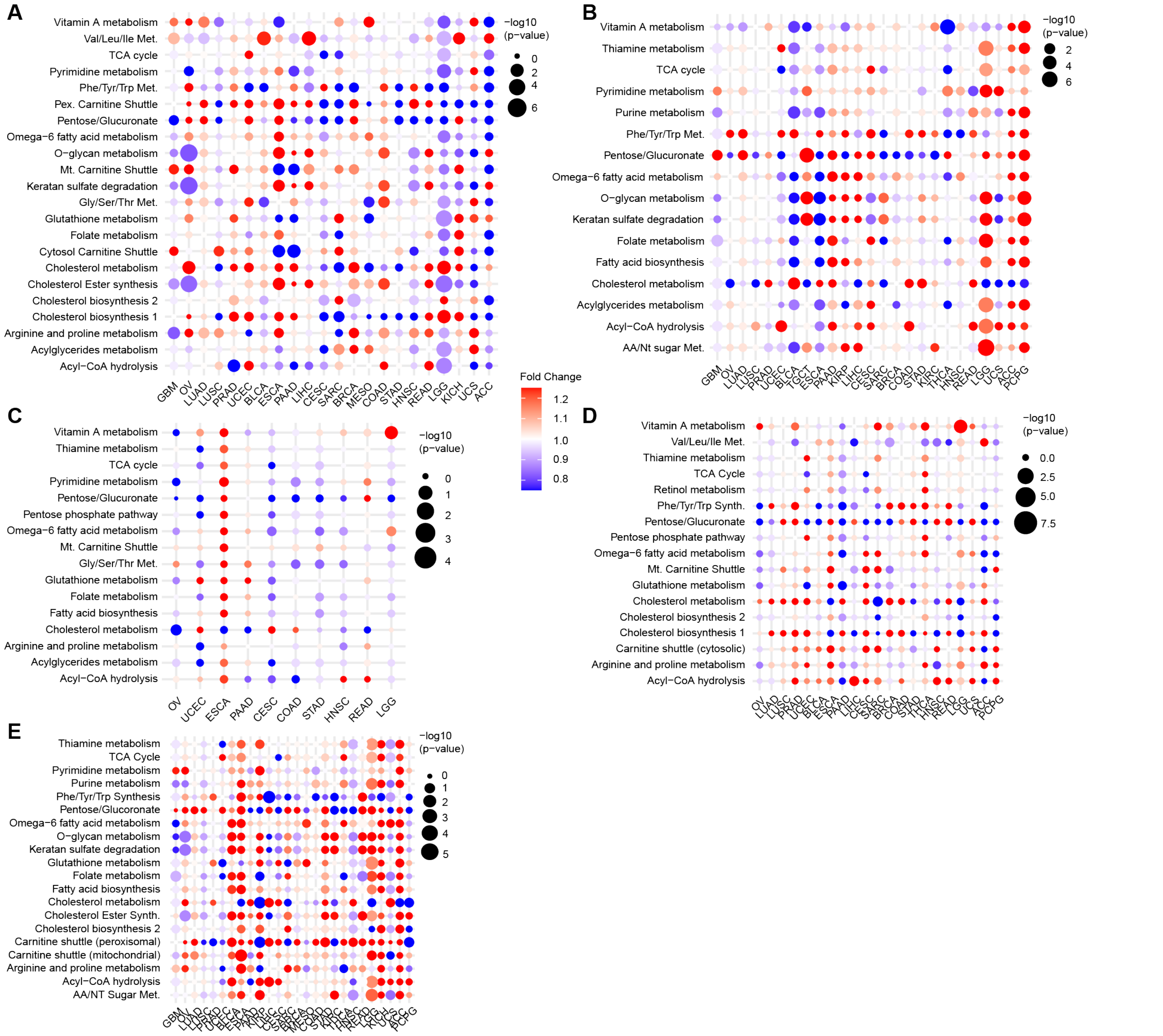

Figure S13: Bubble plots highlighting differences in metabolic flux across tissue types between tumors with and without alterations in:

(**A**) TP53, (**B**) RTK-RAS, (**C**) TGF-Beta, (**D**) Wnt, (**E**) Cell cycle. *P* values were calculated using one-way ANOVA and corrected for multiple hypothesis testing using a Benjamini-Hochberg correction.

Figure S14. Related to Figure 6.

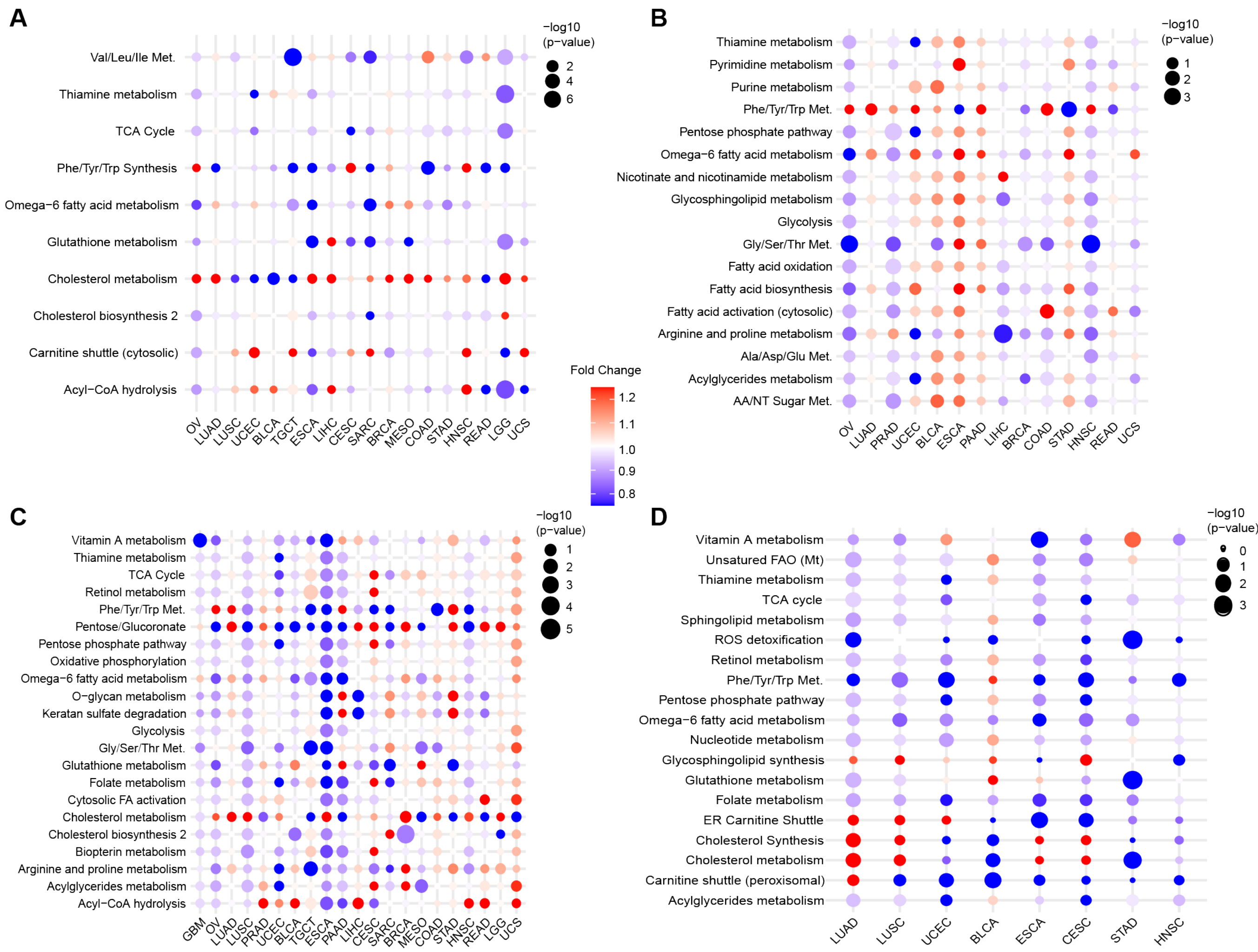

Figure S14: Bubble plots highlighting differences in metabolic flux across tissue types between tumors with and without alterations in:

(**A**) Hippo, (**B**) MYC, (**C**) Notch, and (**D** NRF2. *P* values were calculated using one-way ANOVA and corrected for multiple hypothesis testing using a Benjamini-Hochberg correction.

Figure S15. Related to Figure 7.

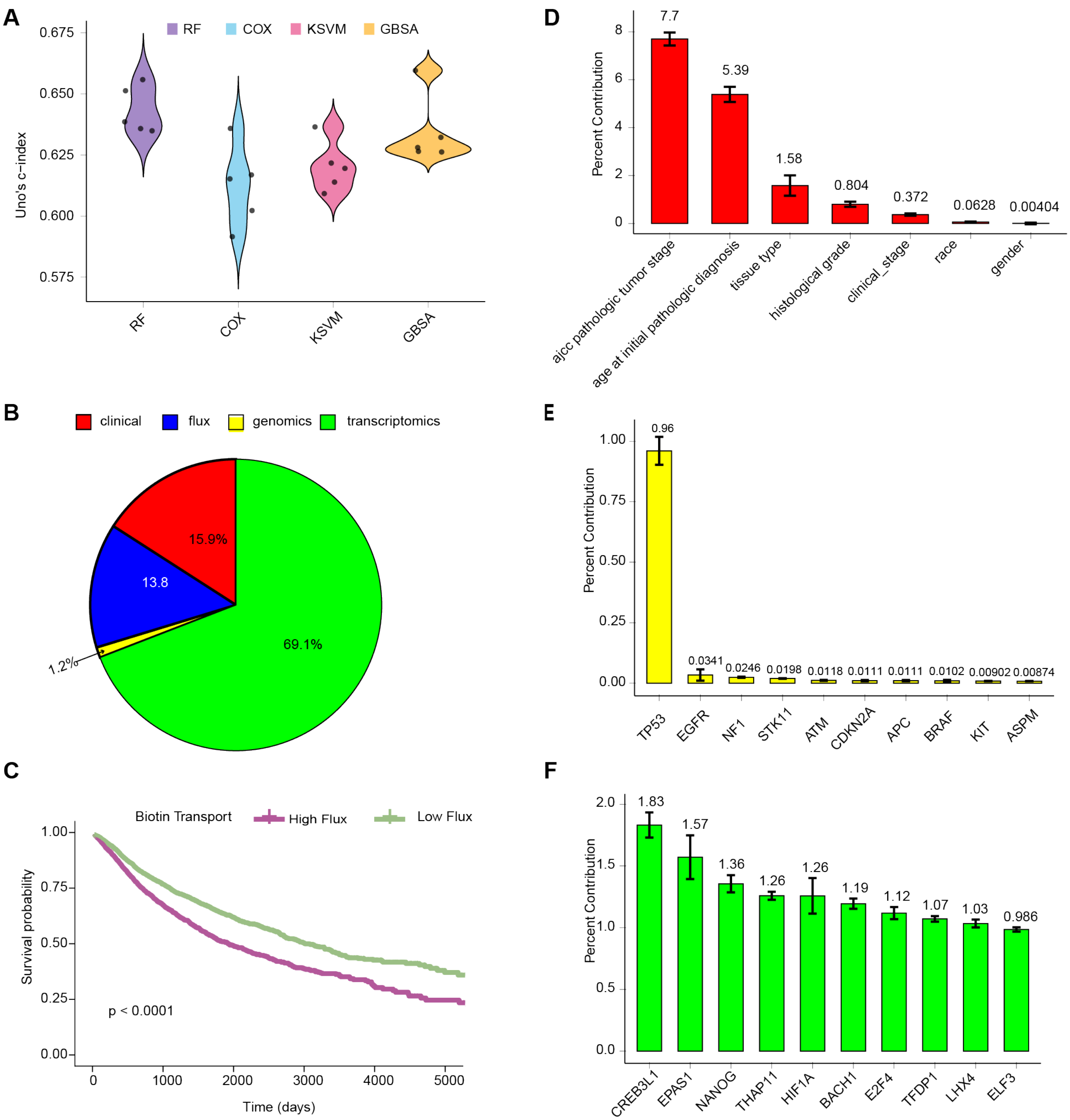

Figure S15: Flux shows predictive power in the ensemble machine learning model.

(**A**) Performance of various algorithms trained on flux data: RF (random survival forest); COX, cox elastic net linear regression models; KSVM, kernel support vector machine; GBSA, gradient boosted survival analysis. RF performed best with flux data, and similar trends were observed for the other omics models. (**B**) Pie chart depicting the contribution of different feature classes to the ensemble model. (**C**) Kaplan-Meier curve of patients stratified by high or low biotin transport flux. (**D**) Barplots of clinical feature importance in the ensemble model. (**E**) Barplots of genomic feature importance in the ensemble model. (**F**) Barplots of transcription factor activity feature importance in the ensemble model. The *P* value in (C) was calculated using a log-rank test.
